# Estimation of Involuntary Components of Human Arm Impedance in Multi-Joint Movements via Feedback Jerk Isolation

**DOI:** 10.3389/fnins.2020.00459

**Published:** 2020-05-25

**Authors:** Hendrik Börner, Satoshi Endo, Sandra Hirche

**Affiliations:** Chair of Information-Oriented Control, Department of Electrical and Computer Engineering, Technical University of Munich, Munich, Germany

**Keywords:** dynamic regressor representation, feedback jerk isolation, human motor control, involuntary impedance components, impedance estimation, human-robot interaction

## Abstract

Stable and efficient coordination in physical human-robot interaction requires consideration of human feedback behavior. In unpredictable tasks, where voluntary cognitive feedback is too slow to guarantee desired task execution, the human must rely on involuntary intrinsic and reflexive feedback. The combined effects of these two feedback mechanisms and the inertial characteristics can be summarized in the involuntary impedance components. In this work, we present a method for the estimation of the involuntary impedance components of the human arm in multi-joint movements. We apply force perturbations to evoke feedback jerks that can be isolated using a high pass filter and limit the duration of the estimation interval to guarantee exclusion of voluntary cognitive feedback. Dynamic regressor representation of the rigid body dynamics of the arm and first order Taylor series expansion of the feedback behavior yield a model that is linear in the involuntary impedance components. The constant values of the inertial parameters are estimated in a static posture maintenance task and subsequently inserted to estimate the remaining components in a dynamic movement task. The method is validated with simulated data of a neuromechanical model of the human arm and its performance is compared to established methods from the literature. The results of the validation demonstrate superior estimation performance for moderate movement velocities, and less influence of the variability of the movements. The applicability to real data and the plausibility of the limited estimation interval are successfully demonstrated in an experiment with human participants.

## 1. Introduction

Technological advancements in robotics are enabling robotic assistance through physical interaction of human and robot for applications in medical, domestic, and industrial domains. In physical human-robot interaction (pHRI), control strategies are designed to provide efficient and intuitive interaction, during which instabilities must be avoided to guarantee safety and comfort of the human. Fulfillment of these requirements requires consideration of human feedback behavior in the control design process.

**Figure 1:**
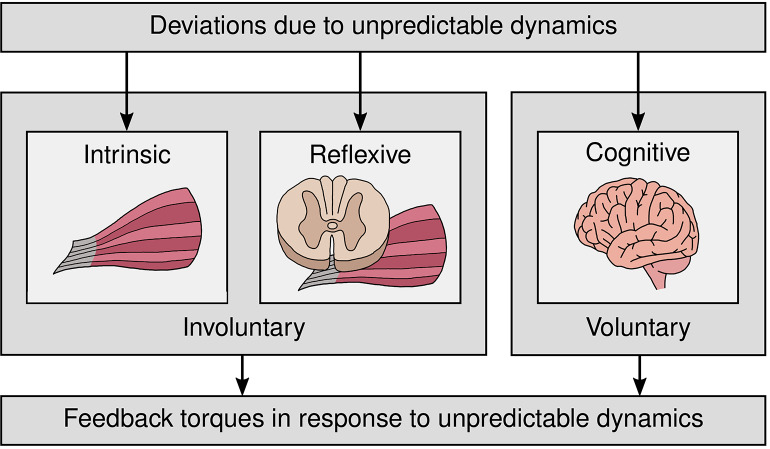
Classification of intrinsic, reflexive, and cognitive feedback due to unpredictable dynamics into involuntary and voluntary feedback. All cognitive feedback at supraspinal level is referred to as voluntary feedback and the combined effects of all feedback mechanisms with shorter delays than voluntary feedback are grouped into involuntary feedback.

During execution of a desired motor task, joint torques produced by the neuromuscular system are composed of a feedforward and a feedback component (Tee et al., 2004). Due to a priori calculation, the feedforward component cannot account for unpredictable dynamics, which can result from incomplete or incorrect internal models (Tomi et al., 2008), inherent neural noise (Faisal et al., 2008), and unexpected external perturbations (Gomi and Kawato, 1997). The resulting deviations are compensated by restoring torques that contain effects of muscle intrinsic viscoelastic properties, reflexive feedback, and cognitive feedback (Gomi and Osu, 1998). Apart from the effects of the muscle intrinsic viscoelastic properties, these torques are produced at different task-dependent delays. As cognitive feedback possesses the longest delays [in the order of 100 ms (Franklin and Wolpert, 2011)], it may be too slow to guarantee desired task execution in unpredictable tasks (Mehta and Schaal, 2002). Thus, in such situations, the human must rely on intrinsic and reflexive feedback. In this work, all cognitive feedback at supraspinal level is referred to as voluntary feedback and the combined effects of all feedback mechanisms with shorter delays than voluntary feedback are grouped into involuntary feedback (see Figure 1).

The restoring torques generated by involuntary feedback can be modeled by a linear system composed of the involuntary impedance components, damping and stiffness (Bennett et al., 1992). Due to length- and velocity-tension relationships of muscle fibers and tendons, both components depend on joint angles and angular velocities. Additionally, the central nervous system (CNS) is able to modulate both components through co-contraction of antagonistic pairs of muscles at the respective joints (Mussa-Ivaldi et al., 1985), e.g., to achieve accuracy requirements (Lametti et al., 2007) or to compensate environmental instabilities (Burdet et al., 2001). Analysis of such modulation strategies in experiments that emulate realistic pHRI may provide valuable information for the control design process, e.g., for stability assessment or control behavior adaptation. However, acquisition of this information requires an estimation method that is compatible with the short estimation interval, in which only involuntary feedback is active. It must enable precise execution of perturbations and accurate isolation of resulting feedback behavior, as estimation errors in the unperturbed states directly influence the estimation accuracy of the involuntary impedance components (Burdet et al., 2013). To the best of the author's knowledge, existing methods are either not able to guarantee exclusion of voluntary feedback (e.g., Gomi and Kawato, 1997; Erden and Billard, 2015) or are limited to application to free, unfettered movements (Piovesan et al., 2013).

**Figure 2:**
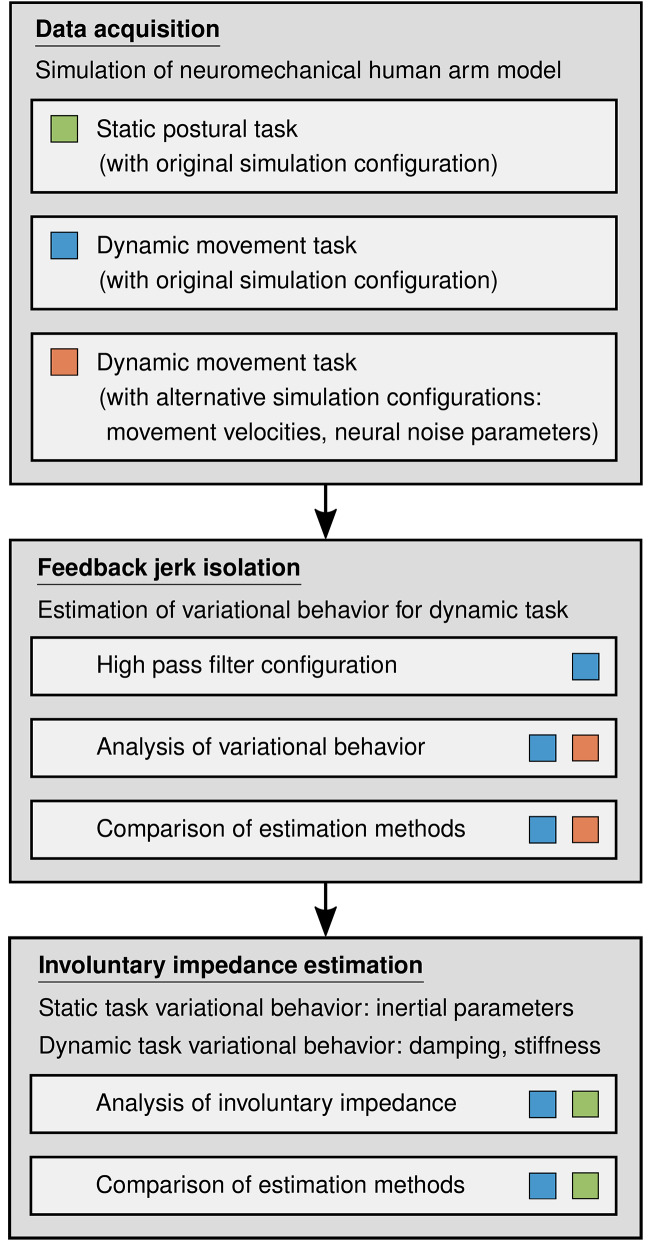
Schematic diagram of the major elements of the method and the validation with simulated data. The colored boxes, which are defined in the data acquisition part of the diagram, indicate which sets of simulated data are applied and analyzed in which phases of the validation.

In this work, we present a method for the estimation of the involuntary impedance components of the human arm in multi-joint movements. We apply force perturbations in order to evoke deviations during two-dimensional point to point arm movements. These perturbations are designed such that the dominant frequencies of the jerks of the evoked feedback behavior lie above those of the unperturbed movements. Thus, the feedback behavior can be isolated by a high pass filter. The duration of the estimation interval is limited to guarantee the exclusion of voluntary feedback. Dynamic regressor representation of the rigid body dynamics and first order Taylor series expansion of the feedback behavior yield a model that is linear in the involuntary impedance components in joint space. The constant values of the inertial parameters are estimated in a static posture maintenance task and subsequently used for the estimation of the remaining involuntary impedance components in a dynamic movement task. Both the feedback jerk isolation and the involuntary impedance estimation are validated with simulated data of a neuromechanical model of the human arm. We compare the validation results to those obtained by application of the methods presented in Gomi and Kawato (1997) and Erden and Billard (2015). In the validation of the feedback jerk isolation, we additionally analyze the effects of different movement velocities as well as different frequencies and amplitudes of neural noise (see Figure 2). Finally, we perform an extensive evaluation of the applicability of the method to real data based on an experiment with human participants. It includes an analysis of the effects of different durations of the estimation interval.

### 1.1. Related Work

Estimation of multi-joint arm impedance is generally either performed by application of a perturbation paradigm, in which perturbations are used to evoke deviations from unperturbed states to measure variational dynamics, or by application of electromyographic (EMG) sensing modalities. In the latter category of approaches, multiple studies combine measurements of muscle activity with parametric muscle models, e.g., linear models of muscle stiffness (Osu and Gomi, 1999), quadratic models of muscle tension (Shin et al., 2009), or calibrated models of musculotendon unit forces (Buzzi et al., 2017). In Kim et al. (2009), an artificial neural network produces a mapping between EMG data and stiffness estimates, that are obtained from measured joint torques and an empirically determined linear model. In Lakatos et al. (2013) and Ajoudani et al. (2015), similar mappings are produced by pairing stiffness estimates obtained by application of the perturbation paradigm with EMG data. All of these EMG approaches have in common that they exclusively estimate stiffness.

Estimation of multi-joint arm impedance by application of the perturbation paradigm can generally be divided into two main categories: estimation in static posture maintenance tasks and in dynamic movement tasks. In both categories, perturbations are used to evoke deviations from unperturbed states and impedance is estimated based on the variational dynamics. Impedance estimation in static posture maintenance tasks is significantly less complex, as it does not require estimation of unperturbed states of an underlying movement. Furthermore, it allows for application of position perturbations, which are executed by moving the hand along a perturbation trajectory and thus enable a priori definition of the variational kinematics. Some studies exploit this advantage by using perturbation trajectories with plateau phases, during which the deviation remains constant (Mussa-Ivaldi et al., 1985; Dolan et al., 1993; Gomi and Osu, 1998; Darainy et al., 2004; Masia and Squeri, 2015). During the plateau phase, variational velocities and accelerations are zero. Thus, the respective variational forces can be used to exclusively estimate stiffness. Some studies use position perturbations defined by third (Artemiadis et al., 2010; Patel et al., 2014) or fifth degree polynomials (Lakatos et al., 2011, 2013), which are specifically designed to improve the conditioning of the estimation. Other studies use stochastic position (Perreault et al., 2004; Ajoudani et al., 2015) or force perturbations (Palazzolo et al., 2007) in combination with significantly longer estimation intervals to estimate frequency domain impedance transfer functions.

Impedance estimation during dynamic movement tasks is significantly more challenging than during static posture maintenance tasks, as it requires precise estimation of the unperturbed states of the underlying movement. In Wang et al. (2001), mechanical impedance is solely estimated in terms of feedback forces. In Hondori and Tech (2011), it is represented by the ratio of frequency domain forces and velocities. In Burdet et al. (2000), position perturbations with plateau phases are used to exclusively estimate stiffness in a 60 ms interval that starts 120 ms after perturbation onset. In Darainy et al. (2007) and Wong et al. (2009b), similar methods are used to exclusively estimate stiffness in a 50 ms interval that starts 250 ms after perturbation onset. In Piovesan et al. (2013), force perturbations and time-frequency analysis are used to estimate impedance 135 ms after perturbation onset. Due to reliance on vibrational energy, this method is only applicable to free, unfettered movements. In Gomi and Kawato (1997), force perturbations are used to estimate impedance by least squares estimation in a 280 ms interval. Erden and Billard (2015) use a similar method to estimate impedance in a 250 ms interval.

To the best of the author's knowledge, among existing methods in the literature, only those presented in Gomi and Kawato (1997) and Erden and Billard (2015) are able to estimate stiffness, damping, and inertial characteristics during multi-joint arm movements that emulate realistic pHRI. In Gomi and Kawato (1997), participants perform transversal and longitudinal point to point movements. The movements are guided by a target position that moves along a reference trajectory and is displayed on a computer monitor. First order Taylor series expansion is used to obtain a linearized model of the rigid body dynamics and the feedback behavior in joint space. Inertial parameters are estimated a priori and used for the estimation of damping and stiffness. Variational dynamics are obtained by calculating the difference to the averaged unbiased dynamics of all perturbed movements, which are calculated by subtracting the offsets at perturbation onset. In Erden and Billard (2015), participants perform a manual tungsten inert gas welding task in cooperation with a robot. The movements are guided by a straight reference trajectory and the participants are instructed “to do their best to achieve a good welding.” Variational behavior is described by a linear model of diagonal inertia, damping, and stiffness matrices in Cartesian space, which are estimated simultaneously. Variational positions and forces are obtained by subtraction of the offsets at perturbation onset and variational velocities and accelerations are obtained by differentiation. Both Gomi and Kawato (1997) and Erden and Billard (2015) are unable to guarantee exclusion of voluntary feedback, as both methods require estimation intervals that are significantly longer than the delay of voluntary feedback. In Gomi and Kawato (1997), this limitation is compensated by application of the do-not-intervene-voluntarily paradigm, which means that participants are instructed to not intervene voluntarily in response to the perturbation. However, this approach poses two problems. Firstly, despite these instructions, voluntary intervention is nonetheless possible and exclusion of voluntary feedback cannot be guaranteed. Secondly, instructing the participants to not intervene voluntarily substantially limits the number of possible, realistic pHRI application scenarios.

A preliminary work has been presented in Börner et al. (2018). In this preliminary work, the rigid body dynamics were linearized in joint space and the involuntary impedance components were estimated in Cartesian space. Due to the linearization in joint space, assumptions concerning negligibility of inertia and Coriolis terms were necessary to obtain a linear second order system in Cartesian space. The elements of the Cartesian endpoint inertia were calculated with anthropometric data and subsequently inserted to estimate the remaining involuntary impedance components in a dynamic movement task. Furthermore, the application of a preliminary perturbation design required assumptions regarding correlations of the beginning of the estimation interval and noticeable deviations due to the perturbation.

The remainder of this article is structured as follows: section 2 contains the materials and methods, which include the derivation of the involuntary impedance model as well as the presentation of the feedback jerk isolation, the perturbation design, and the involuntary impedance estimation. Furthermore, the details of the simulation and the experiment are presented. Section 3 contains the results, which include the validation and the comparison with existing methods based on simulated data as well as the evaluation with experimental data. Section 4 contains the discussion and conclusions are summarized in section 5.

## 2. Materials and Methods

We begin this section by formulating the problem considered in this work. Subsequently, we present the feedback jerk isolation and the involuntary impedance estimation. Finally, we introduce the details of the simulation and the experiment.

### 2.1. Problem Formulation

In this section, we first derive a general human motor behavior model, which then serves as the basis for the derivation of a model of the involuntary impedance components.

#### 2.1.1. Human Motor Behavior Model

The human arm is modeled as a multi-joint two-link system. In order to reduce complexity and neglect gravity, movements are constrained to the horizontal plane. The rigid body dynamics is given by

(1) $$\begin{array}{l} \tau_{\text{int}}+\tau_{\text{ext}}=M_{q}\left(q\right)\ddot{q}+C\left(q,\dot{q}\right)\dot{q}\;, \end{array}$$

where ***q*** is the 2 degree of freedom (DoF) arm configuration in joint space, *M*_*q*_ is the symmetric and positive-definite inertia matrix (Hogan, 1985), *C* is the Coriolis/centrifugal matrix, ***τ***_int_ are the internally generated joint torques, and ***τ***_ext_ are the external torques (Gomi and Kawato, 1997). The arm configuration $q={\left[q_{1},q_{2}\right]}^{\text{T}}$ is defined by the angles of the shoulder *q*_1_ and the elbow *q*_2_.

The internal torques ***τ***_int_ are produced by the muscle tensions ***m*** that act on the musculoskeletal system:

(2) $$\begin{array}{l} \tau_{\text{int}}=J_{m}^{\text{T}}\left(\lambda \right)m\left(\lambda ,\dot{\lambda },a\right)\;, \end{array}$$

where ***λ*** are the muscle lengths, $\dot{\lambda }$ are the respective derivatives, ***a*** are the muscle activations, and *J*_*m*_ is the muscle Jacobian that contains the muscle moment arms that are necessary for the transformation to joint space (Shin et al., 2009). During execution of a motor task, the muscle activations ***a*** consist of a feedforward term ***a***_FF_, a feedback term ***a***_FB_, and a neural noise term ***a***_N_ (Franklin et al., 2008):

(3) $$\begin{array}{l} a=a_{\text{FF}}\left(\theta \right)+a_{\text{FB}}+a_{\text{N}}\left(a\right)\;. \end{array}$$

The feedforward term ***a***_FF_ is calculated by the CNS through a priori optimization of the statistics of the desired motor behavior with respect to costs defined by the task-specific input parameters ***θ***, which depend on factors, such as environmental constraints and task requirements (Harris and Wolpert, 1998). The neural noise term ***a***_N_ represents signal-dependent noise whose variance increases with the magnitude of the muscle activations ***a*** (Slifkin and Newell, 1999). Deviations caused by unpredictable dynamics are compensated by the feedback term ***a***_FB_, which consists of multiple components that depend on delayed afferent sensory information and are produced at different delays (Franklin and Wolpert, 2011):

(4) $$a_{\text{FB}}=\{\begin{array}{ll} 0 & \forall \;t_{\text{p}}\in \left[0,\delta_{\text{r}}\right]\\ a_{\text{FB},\text{r}}(\lambda ,\dot{\lambda }) & \forall \;t_{\text{p}}\in ]\delta_{\text{r}},\delta_{\text{v}}]\\ a_{\text{FB},\text{r}}(\lambda ,\dot{\lambda })+a_{\text{FB},\text{v}}(\theta ) & \forall \;t_{\text{p}}>\delta_{\text{v}} \end{array}\;,$$

where ***a***_FB,r_ and ***a***_FB,v_ are reflexive and voluntary feedback muscle activations, respectively. The variables δ_r_ and δ_v_ are the corresponding delays and *t*_p_ = *t* − *t*_0_ is the time after the onset of the unpredictable dynamics at *t*_0_. In the interval ]δ_r_, δ_v_], the feedback muscle activations ***a***_FB_ only consist of reflexive feedback muscle activations ***a***_FB,r_, which are affected by neural conduction delays that depend on the length and type of the nerve fiber. The fastest reflexive feedback is produced by the short-latency monosynaptic stretch reflex, with a delay δ_r,s_ in the order of 10 − 40 ms (Matthews, 1991). Slightly slower reflexive feedback is produced by the cortical component of the long-latency stretch reflex, with a delay δ_r,l_ in the order of 30 − 70 ms (Thilmann et al., 1991). As the feedback behavior in this interval does not recruit higher-order cognitive processes, the reflexive feedback muscle activations ***a***_FB,r_ only depend on the muscle lengths ***λ*** and the respective derivatives $\dot{\lambda }$. For all *t*_p_ > δ_v_, the voluntary feedback muscle activations ***a***_FB,v_, which are defined by the task-specific input parameters ***θ***, additionally contribute to the feedback muscle activations ***a***_FB_. For voluntary feedback in response to haptic motion perception, the transmission of the associated proprioceptive sensory information to the CNS is subject to delays in the order of 100 ms. Conduction delays of descending motor commands from the motor cortex to the arm muscles are ~ 15 ms (Merton and Morton, 1980). Consequently, the minimum delay δ_v_ of the voluntary feedback muscle activations ***a***_FB,v_ is in the order of 115 ms.

The internal torques ***τ***_int_ are composed of a feedforward term ***τ***_FF_, a feedback term ***τ***_FB_, and a neural noise term ***τ***_N_, that represent the equivalents to the respective muscle activations ***a*** in (3):

(5) $$\begin{array}{l} \tau_{\text{int}}=\tau_{\text{FF}}\left(\theta \right)+\tau_{\text{FB}}+\tau_{\text{N}}\left(a\right). \end{array}$$

Analogous to the feedback muscle activations ***a***_FB_ in (4), the feedback torques ***τ***_FB_ consist of multiple components that are produced at different delays (Lakatos et al., 2011):

(6) $$\tau_{\text{FB}}=\{\begin{array}{ll} \tau_{\text{FB},\text{i}} & \forall \;t_{\text{p}}\in \left[0,\delta_{\text{r}}\right]\\ \tau_{\text{FB},\text{i}}+\tau_{\text{FB},\text{r}} & \forall \;t_{\text{p}}\in ]\delta_{\text{r}},\delta_{\text{v}}]\\ \tau_{\text{FB},\text{i}}+\tau_{\text{FB},\text{r}}+\tau_{\text{FB},\text{v}} & \forall \;t_{\text{p}}>\delta_{\text{v}} \end{array}\;,$$

where ***τ***_FB,i_, ***τ***_FB,r_, and ***τ***_FB,v_ are intrinsic, reflexive, and voluntary feedback torques, respectively. In the interval [0, δ_r_], the feedback torques ***τ***_FB_ only consist of intrinsic feedback torques ***τ***_FB,i_, which result from muscle intrinsic viscoelastic properties and are thus not affected by any delays (Tee et al., 2004). In the subsequent interval ]δ_r_, δ_v_] and for all *t*_p_ > δ_v_, the feedback torques ***τ***_FB_ additionally contain contributions from reflexive feedback torques ***τ***_FB,r_ and voluntary feedback torques ***τ***_FB,v_, respectively. Inserting (6) in (5) yields the internal torques ***τ***_int_ for all *t* > δ_v_:

(7) $$\begin{array}{l} \tau_{\text{int}}=\tau_{\text{FF}}\left(\theta \right)+\tau_{\text{FB},\text{i}}+\tau_{\text{FB},\text{r}}+\tau_{\text{FB},\text{v}}+\tau_{\text{N}}\left(a\right)\;. \end{array}$$

This general model represents the basis for the derivation of the involuntary impedance model.

#### 2.1.2. Dynamic Regressor Representation

The rigid body dynamics (1) is non-linear with respect to the arm configuration ***q*** and the respective derivatives $\dot{q}$ and $\ddot{q}$. Nonetheless, the Lagrangian formalism enables the derivation of the dynamic regressor representation, which is linear with respect to the standard inertial parameters of the system:

(8) $$\begin{array}{l} \tau_{\text{int}}+\tau_{\text{ext}}=Y\left(q,\dot{q},\ddot{q}\right)\pi \;, \end{array}$$

(9) $$\begin{array}{l} Y\left(q,\dot{q},\ddot{q}\right)=\frac{\partial \left(M_{q}\left(q\right)\ddot{q}+C\left(q,\dot{q}\right)\dot{q}\right)}{\partial \pi }\;, \end{array}$$

where *Y* is the regressor matrix and $\pi ={\left[\pi_{1}^{\text{T}},\pi_{2}^{\text{T}}\right]}^{\text{T}}$ is the standard inertial parameter vector, which is composed of the inertial parameters of the upper arm ***π***_1_ and the forearm ***π***_2_ (Gautier and Khalil, 1988). In general, the inertial parameters consist of the masses and the mass moments of first and second order (Khalil and Dombre, 2004). However, some parameters do not have any effect on the system dynamics which corresponds to a zero column in the regressor matrix *Y*. For the multi-joint arm, omission of these parameters yields the reduced inertial parameter vector

(10) $$\begin{array}{l} \pi_{\text{r}}={\left[I_{x_{2}x_{2},1},\;I_{x_{2}x_{2},2},\;m_{2}l_{cx_{3},2},\;m_{2}\right]}^{\text{T}}\;, \end{array}$$

in which, according to the parallel axis theorem,

(11) $$\begin{array}{l} I_{x_{2}x_{2},i}={\tilde{I}}_{x_{2}x_{2},i}+m_{i}l_{cx_{3},i}^{2}\;. \end{array}$$

Thus, the inertial properties are defined by the moments of inertia *Ĩ*_*x*_2_*x*_2_, *i*_, the masses *m*_*i*_, and the lengths from the joints to the centers of gravity of the links *l*_*c*_*x*__3_, *i*_. Comprehensive analysis of the reduced regressor matrix *Y*_r_ reveals that further parameter reduction is possible. Appropriate transformations yield the base inertial parameter (BIP) vector $\bar{\pi }$, which contains the minimal set of parameters, and the corresponding base regressor matrix $\bar{Y}$ of the multi-joint arm:

(12) $$\begin{array}{l} \bar{\pi }={\left[I_{x_{2}x_{2},1}+m_{2}l_{1}^{2},\;I_{x_{2}x_{2},2},\;m_{2}l_{cx_{3},2}l_{1}\right]}^{\text{T}}\;, \end{array}$$

(13) $$\begin{array}{l} \bar{Y}\left(q,\dot{q},\ddot{q}\right)=\left[\begin{array}{ccc} {\ddot{q}}_{1} & {\ddot{q}}_{1}+{\ddot{q}}_{2} & {\bar{y}}_{1,3}\\ 0 & {\ddot{q}}_{1}+{\ddot{q}}_{2} & {\bar{y}}_{2,3} \end{array}\right]\;, \end{array}$$

with

$$\begin{array}{l} {\bar{y}}_{1,3}=\left(2{\ddot{q}}_{1}+{\ddot{q}}_{2}\right)\text{cos}\left(q_{2}\right)-\left(2{\dot{q}}_{1}{\dot{q}}_{2}+{\dot{q}}_{2}^{2}\right)\text{sin}\left(q_{2}\right)\;,\\ {\bar{y}}_{2,3}={\ddot{q}}_{1}\text{cos}\left(q_{2}\right)+{\dot{q}}_{1}^{2}\text{sin}\left(q_{2}\right)\;, \end{array}$$

where *l*_1_ is the length of the upper arm (Klodmann et al., 2015).

#### 2.1.3. Involuntary Impedance Model

While the sum of the intrinsic feedback torques ***τ***_FB,i_ and the reflexive feedback torques ***τ***_FB,r_ can be modeled by a linear system composed of the involuntary impedance components, damping and stiffness (Bennett et al., 1992), the voluntary feedback torques ***τ***_FB,v_ are highly task-specific and can therefore not be modeled by such a general formulation (Franklin and Wolpert, 2011). Therefore, in this work, in order to guarantee exclusion of voluntary feedback, we limit the duration of the estimation interval to *T*_est_ = δ_v_. Due to the short latency of the reflexive feedback and the variability of the respective delays, separation of the intrinsic and the reflexive feedback contributions is difficult (Burdet et al., 2013). Thus, we summate the contributions in the involuntary feedback torques

(14) $${\bar{\tau }}_{\text{FB}}=\{\begin{array}{ll} \tau_{\text{FB},\text{i}} & \forall \;t_{\text{p}}\in \left[0,\delta_{\text{r}}\right]\\ \tau_{\text{FB},\text{i}}+\tau_{\text{FB},\text{r}} & \forall \;t_{\text{p}}\in ]\delta_{\text{r}},T_{\text{est}}] \end{array}\;.$$

For small deviations, the feedback behavior evoked by the force perturbations can be described by a linearized model obtained by first order Taylor series expansion of the general model (7) about the unperturbed states q^*^, ${\dot{q}}^{*}$, ${\ddot{q}}^{*}$, a^*^, $\tau_{\text{int}}^{*}$, and $\tau_{\text{ext}}^{*}$. According to (2), the variational internal torques $\Delta \tau_{\text{int}}=\tau_{\text{int}}^{*}-\tau_{\text{int}}$ after the onset of the perturbation depend on the muscle lengths **λ**, the respective derivatives $\dot{\lambda }$, and the muscle activations ***a***. Due to the confinement to the estimation interval [0, *T*_est_], the variational muscle activations Δ***a*** = a^*^ − ***a***, as defined by (3) and (4), can only consist of reflexive feedback muscle activations ***a***_FB,r_. Considering $\Delta a=f\left(\lambda ,\dot{\lambda }\right)$ as well as **λ** = *J*_*m*_(**λ**)***q*** and $\dot{\lambda }=J_{m}\left(\lambda \right)\dot{q}$, linearization of the internal torques ***τ***_int_ for *t*_p_ ∈ [0, *T*_est_] yields

(15) $$\begin{array}{l} \Delta \tau_{\text{int}}=\frac{\text{d}\tau_{\text{int}}}{\text{d}\dot{q}}\Delta \dot{q}+\frac{\text{d}\tau_{\text{int}}}{\text{d}q}\Delta q\;, \end{array}$$

in which all variational variables, indicated by a Δ symbol, represent the deviations from the unperturbed states, e.g., Δ***q*** = q^*^ − ***q***, and the total derivatives are defined as

(16) $$\begin{array}{l} \frac{\text{d}\tau_{\text{int}}}{\text{d}\dot{q}}=\frac{\partial \tau_{\text{int}}}{\partial \dot{q}}+\frac{\partial \tau_{\text{int}}}{\partial a}\frac{\partial a}{\partial \dot{q}}\;,\;\frac{\text{d}\tau_{\text{int}}}{\text{d}q}=\frac{\partial \tau_{\text{int}}}{\partial q}+\frac{\partial \tau_{\text{int}}}{\partial a}\frac{\partial a}{\partial q}\;. \end{array}$$

Inserting (7) for *t*_p_ ∈ [0, *T*_est_] and (14) in (15) yields

(17) $$\begin{array}{l} \Delta \tau_{\text{int}}=-D_{q}\left(q,\dot{q},a\right)\Delta \dot{q}-K_{q}\left(q,\dot{q},a\right)\Delta q\;. \end{array}$$

In this linearized model, the involuntary joint damping *D*_*q*_ and the involuntary joint stiffness *K*_*q*_, which are both part of the involuntary impedance components^1^, are defined as

(18) $$\begin{array}{l} D_{q}\left(q,\dot{q},a\right)=-\frac{\text{d}{\bar{\tau }}_{\text{FB}}}{\text{d}\dot{q}}\;,\;K_{q}\left(q,\dot{q},a\right)=-\frac{\text{d}{\bar{\tau }}_{\text{FB}}}{\text{d}q}\;. \end{array}$$

Inserting the BIP vector $\bar{\pi }$ of (12) and the base regressor matrix $\bar{Y}$ of (13) in (8) and using the resulting linear model to calculate the variational internal torques Δ***τ***_int_ yields

(19) $$\begin{array}{l} \Delta \tau_{\text{int}}=\left(\bar{Y}\left(q^{*},{\dot{q}}^{*},{\ddot{q}}^{*}\right)-\bar{Y}\left(q,\dot{q},\ddot{q}\right)\right)\bar{\pi }-\Delta \tau_{\text{ext}}\\ \;=\Delta \bar{Y}\left(q,q^{*},\dot{q},{\dot{q}}^{*},\ddot{q},{\ddot{q}}^{*}\right)\bar{\pi }-\Delta \tau_{\text{ext}}\;. \end{array}$$

Subsequently inserting (17) in (19) yields the involuntary impedance model

(20) $$\begin{array}{l} \Delta \tau_{\text{ext}}=\Delta \bar{Y}\left(q,q^{*},\dot{q},{\dot{q}}^{*},\ddot{q},{\ddot{q}}^{*}\right)\bar{\pi }\\ \;+D_{q}\left(q,\dot{q},a\right)\Delta \dot{q}+K_{q}\left(q,\dot{q},a\right)\Delta q\;. \end{array}$$

In some pHRI scenarios, measurements are performed in Cartesian space. In these scenarios, the arm configuration ***q*** can be obtained from the arm endpoint configuration ***x*** with the inverse kinematics

(21) $$\begin{array}{l} q_{1}=\text{ta}{\text{n}}^{-1}\left(\frac{x_{2}}{x_{1}}\right)-\text{ta}{\text{n}}^{-1}\left(\frac{l_{2}\text{sin}\left(q_{2}\right)}{l_{1}+l_{2}\text{cos}\left(q_{2}\right)}\right)\;, \end{array}$$

(22) $$\begin{array}{l} q_{2}=\text{co}{\text{s}}^{-1}\left(\frac{x_{1}^{2}+x_{2}^{2}-l_{1}^{2}-l_{2}^{2}}{2l_{1}l_{2}}\right)\;, \end{array}$$

where *l*_2_ is the length of the forearm and the Cartesian origin is located in the shoulder joint. Furthermore, the Jacobian *J* can be used to transform the external endpoint forces ***u***_ext_ to the external torques

(23) $$\begin{array}{l} \tau_{\text{ext}}=J^{\text{T}}\left(q\right)u_{\text{ext}}\;. \end{array}$$

The problem considered in this work consists of the estimation of the involuntary impedance components, i.e., BIP vector $\bar{\pi }$, damping *D*_*q*_, and stiffness *K*_*q*_, in the estimation interval [0, *T*_est_]. This is to be achieved given the perturbed observations {***x***, ***u***_ext_}, which result from application of force perturbations during multi-joint arm movements, and requires estimation of the unperturbed dynamics $\left\{q^{*},{\dot{q}}^{*},{\ddot{q}}^{*},\tau_{\text{ext}}^{*}\right\}$.

### 2.2. Involuntary Impedance Estimation

In this section, we first present the estimation of the unperturbed dynamics via feedback jerk isolation and the perturbation design. Subsequently, we present the involuntary impedance estimation.

#### 2.2.1. Feedback Jerk Isolation

According to the minimum jerk principle (Flash and Hogan, 1985), the CNS optimizes the arm endpoint trajectory in a point to point movement through minimization of the total endpoint jerk. We take advantage of this by designing the perturbation such that the dominant frequencies of the jerks of the evoked feedback behavior lie above those of the unperturbed movements. Due to this design, we are able to estimate the evoked feedback behavior in the form of the variational jerks $\Delta \overset{.}{x}$ through application of a high pass filter to the jerks $\overset{.}{x}$ of the perturbed movement. In order to achieve maximum pass band flatness and fast stop band roll-off, we perform this feedback jerk isolation with a Butterworth filter that is implemented as a digital biquad filter with filter order *n*_HP_ = 10. It is applied bi-directionally for zero phase distortion and the cut-off frequencies ***f***_c,HP_ are defined based on the energy spectral densities (ESDs) ***ψ*** of the jerks $\overset{.}{x}$ of the unperturbed and perturbed movements (Stein, 2000):

(24) $$\begin{array}{l} \psi \left(f\right)=|\int_{-\infty }^{\infty }e^{-2\pi ift}\dddot{x}\left(t\right)dt{|}^{2}\;. \end{array}$$

We use the set Ψ, which contains the ESDs ***ψ*** of the jerks $\overset{.}{x}$ of all unperturbed and perturbed movements, to calculate the averaged ESDs of the unperturbed movements ***ψ***_UP_, the perturbed movements ***ψ***_P_, and the resulting feedback behavior ***ψ***_FB_ = ***ψ***_P_ − ***ψ***_UP_. The cut-off frequencies ***f***_c,HP_ are defined as the frequencies ***f*** at which ***ψ***_FB_ > ***ψ***_UP_, i.e., the averaged ESDs of the feedback behavior ***ψ***_FB_ become larger than those of the unperturbed movements ***ψ***_UP_. The high pass filtered jerks ${\overset{⃛}{x}}_{\text{HP}}$ yield the estimated variational jerks $\Delta \overset{⃛}{\hat{x}}$ and the estimated unperturbed jerks ${\overset{⃛}{\hat{x}}}^{*}$. Integration then yields the estimated variational kinematics $\{\Delta \hat{x},\Delta \dot{\hat{x}},\Delta \ddot{\hat{x}}stretchy='false'\}$ and the estimated unperturbed kinematics $\left\{{\hat{x}}^{*},{\dot{\hat{x}}}^{*},{\ddot{\hat{x}}}^{*}\right\}$. For simplicity, from this point on, we refer to the averaged ESDs ***ψ***_UP_, ***ψ***_P_, and ***ψ***_FB_ simply as ESDs.

**Figure 3:**
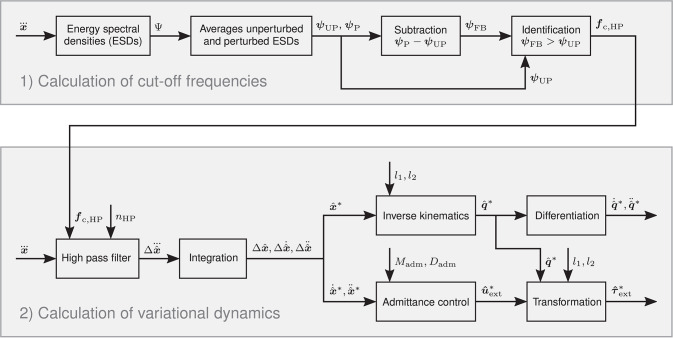
Block diagram of the feedback jerk isolation. For every variational variable Δχ, the respective unperturbed variable χ^*^ = Δχ + χ is calculated with the respective perturbed variable χ, and vice versa.

The apparatus in our experiments is controlled by means of an admittance control scheme. Therefore, the external forces ***u***_ext_ can be calculated with

(25) $$\begin{array}{l} u_{\text{ext}}=u_{\text{pert}}-u_{\text{adm}}-M_{\text{handle}}\ddot{x}\;, \end{array}$$

(26) $$\begin{array}{l} u_{\text{adm}}=M_{\text{adm}}\ddot{x}+D_{\text{adm}}\dot{x}\;, \end{array}$$

where ***u***_pert_ is the perturbation force, ***u***_adm_ is the admittance force, *M*_handle_ is the handle inertia, and *M*_adm_ and *D*_adm_ are the admittance inertia and damping, respectively. Inserting the estimated unperturbed kinematics ${\dot{\hat{x}}}^{*}$ and ${\ddot{\hat{x}}}^{*}$ in (25) and (26) yields the estimated unperturbed external forces ${û}_{\text{ext}}^{*}$ and the estimated variational external forces $\Delta {\hat{u}}_{\text{ext}}$. The estimated unperturbed arm configuration ${\hat{q}}^{*}$ and the respective derivatives ${\dot{\hat{q}}}^{*}$ and ${\ddot{\hat{q}}}^{*}$ are calculated using the inverse kinematics (21) and (22) and the estimated unperturbed external torques ${\hat{\tau }}_{\text{ext}}^{*}$ are calculated using the transformation (23). Finally, the estimated unperturbed dynamics $\left\{{\hat{q}}^{*},{\dot{\hat{q}}}^{*},{\ddot{\hat{q}}}^{*},{\hat{\tau }}_{\text{ext}}^{*}\right\}$ yield the estimated variational dynamics $\left\{\Delta \hat{q},\Delta \dot{\hat{q}},\Delta \ddot{\hat{q}},\Delta {\hat{\tau }}_{\text{ext}}\right\}$. Figure 3 presents a block diagram that illustrates the complete procedure of the feedback jerk isolation.

#### 2.2.2. Perturbation Design

**Figure 4:**
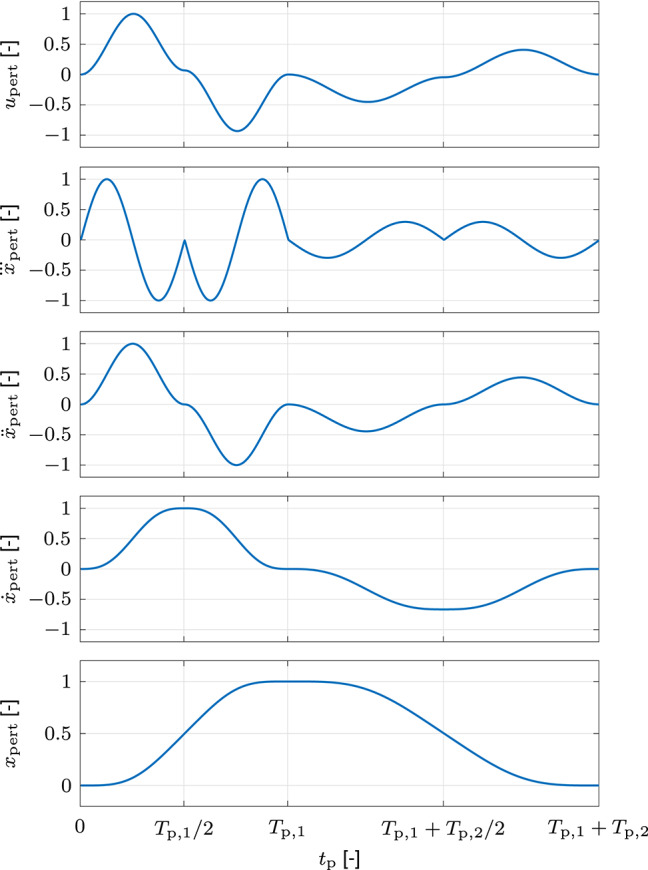
Normalized perturbation force profile *u*_pert_ and kinematics profiles $\left\{x_{\text{pert}},{\overset{.}{x}}_{\text{pert}},{\overset{¨}{x}}_{\text{pert}},{\overset{⃛}{x}}_{\text{pert}}\right\}$ of the dynamic movement task in the simulation and the experiment. The first part of the perturbation with duration *T*_p,1_ is responsible for the deviation from the unperturbed states and the second part of the perturbation with duration *T*_p,2_ supplies the retracting movement toward the unperturbed states.

The perturbation is designed to meet two essential criteria. First, the dominant frequencies of the jerks of the evoked feedback behavior should lie above those of the unperturbed movements. Second, in order to minimize movement interference and corrective oscillations, it should not only produce deviations, but also move the hand back toward the unperturbed states. In order to fulfill these criteria, we design the perturbation acceleration profile *ẍ*_pert_ through concatenation of two sinusoidal functions

(27) $$\xi_{\text{p}}=\{\begin{array}{ll} \frac{1}{2}A_{\text{p}}\left(\xi_{\text{p},\text{sin}}+1\right) & \forall \;t_{\text{p}}\in \;[0,\frac{T_{\text{p}}}{2}]\\ -\frac{1}{2}A_{\text{p}}\left(\xi_{\text{p},\text{sin}}+1\right) & \forall \;t_{\text{p}}\in [\frac{T_{\text{p}}}{2},T_{\text{p}}] \end{array}$$

with

(28) $$\begin{array}{l} \xi_{\text{p},\text{sin}}=\sin\left(\frac{4\pi t_{\text{p}}}{T_{\text{p}}}+\frac{3\pi }{2}\right)\;, \end{array}$$

where *A*_p_ and *T*_p_ are amplitude and duration. The first of the two functions ξ_p,1_ with amplitude *A*_p,1_ and duration *T*_p,1_ is responsible for the deviation from the unperturbed states. The second function ξ_p,2_ with amplitude *A*_p,2_ and duration *T*_p,2_ supplies the retracting movement toward the unperturbed states. In order to minimize hardware oscillations due to the perturbation, we define *T*_p,2_ = (3/2)*T*_p,1_ and scale *A*_p,2_ such that all perturbation profiles are zero at the end of the perturbation. Lastly, we combine the perturbation acceleration profile *ẍ*_pert_ and velocity profile ẋ_pert_ with the admittance inertia *M*_adm_ and damping *D*_adm_ of our apparatus to obtain the perturbation force profile *u*_pert_. It is defined by the amplitude of its first peak *A*_pert_ and its duration *T*_pert_ = *T*_p,1_ + *T*_p,2_. In the dynamic movement task in this work, *A*_pert_ = 40 N and *T*_p,1_ = 70 ms result in *T*_pert_ = 175 ms. The resulting normalized perturbation force profile *u*_pert_ and the corresponding kinematics profiles $\left\{x_{\text{pert}},{\overset{.}{x}}_{\text{pert}},{\overset{¨}{x}}_{\text{pert}},{\overset{.}{x}}_{\text{pert}}\right\}$ are illustrated in Figure 4. Given the normalized perturbation force profile *u*_pert_, the perturbation force

(29) $$\begin{array}{l} u_{\text{pert}}=A_{\text{pert}}u_{\text{pert}}{\left[\cos\phi_{\text{pert}},\sin\phi_{\text{pert}}\right]}^{\text{T}}\;, \end{array}$$

in which the perturbation angle ϕ_pert_ is defined by the set of perturbation angles Φ_pert_.

#### 2.2.3. Involuntary Impedance Estimation

Due to the limited duration of the estimation interval *T*_est_, we assume that the involuntary impedance components *D*_*q*_ and *K*_*q*_ are constant for *t*_p_ ∈ [0, *T*_est_]. Concatenation of the elements of the BIP vector $\bar{\pi }$ and the resulting involuntary impedance parameters ${\bar{D}}_{q}$ and ${\bar{K}}_{q}$ in the unknown model parameter vector

(30) $$\begin{array}{r} \zeta =[{\bar{\pi }}_{1},{\bar{\pi }}_{2},{\bar{\pi }}_{3},{\bar{D}}_{q,11},{\bar{D}}_{q,12},{\bar{D}}_{q,21},{\bar{D}}_{q,22},\\ {{\bar{K}}_{q,11},{\bar{K}}_{q,12},{\bar{K}}_{q,21},{\bar{K}}_{q,22}]}^{\text{T}} \end{array}$$

allows expression of (20) by the identification model

(31) $$\begin{array}{l} A\zeta =b\;, \end{array}$$

where *A* is the observation matrix and ***b*** is the output vector. For an interval with a total of *N* samples

(32) $$\begin{array}{l} A={\left[X^{\text{T}}\left(1\right),X^{\text{T}}\left(2\right),\;\ldots \;,X^{\text{T}}\left(N\right)\right]}^{\text{T}}\;, \end{array}$$

(33) $$\begin{array}{l} b={\left[\Delta {\hat{\tau }}_{\text{ext}}^{\text{T}}\left(1\right),\Delta {\hat{\tau }}_{\text{ext}}^{\text{T}}\left(2\right),\;\ldots \;,\Delta {\hat{\tau }}_{\text{ext}}^{\text{T}}\left(N\right)\right]}^{\text{T}}\;, \end{array}$$

in which the independent variable matrix *X* is defined as

(34) $$\begin{array}{l} X=\frac{\partial \left(\Delta \bar{Y}\left(q,{\hat{q}}^{*},\dot{q},{\dot{\hat{q}}}^{*},\ddot{q},{\ddot{\hat{q}}}^{*}\right)\hat{\bar{\pi }}+{\bar{D}}_{q}\Delta \dot{\hat{q}}+{\bar{K}}_{q}\Delta \hat{q}\right)}{\partial \zeta }\;. \end{array}$$

The estimated model parameter vector $\hat{\zeta }$ is given by

(35) $$\begin{array}{l} \hat{\zeta }={\left(A^{\text{T}}A\right)}^{-1}A^{\text{T}}b\;. \end{array}$$

Due to the limited duration of the estimation interval *T*_est_ and the likewise limited duration of the perturbation *T*_pert_, the perturbation in (29) does not possess sufficient richness of frequency components to guarantee persistent excitation (Söderström and Stoica, 1989). A common approach for the compensation of such effects is the a priori estimation of the inertial parameters (Gomi and Kawato, 1997; Darainy et al., 2007; Wong et al., 2009a,b) which only marginally influences the estimated impedance parameters (Gomi and Osu, 1998). As the inertial parameters possess constant values that are independent of the dynamics, the elements of the BIP vector $\bar{\pi }$ can be estimated a priori in a static postural task.

The intrinsic feedback behavior of the multi-joint arm is governed by spring-like characteristics that result from the elastic properties of the individual muscles. Thus, the intrinsic feedback forces possess zero curl and the intrinsic stiffness is symmetric (Shadmehr and Arbib, 1992). The reflexive feedback forces may possess non-zero curl which can only result from heteronymous inter-muscular reflex arcs with unequal activation gains (Hogan, 1985). However, as the resulting antisymmetric stiffness elements are significantly smaller than the corresponding symmetric ones, the reflexive feedback behavior is nonetheless governed by predominantly spring-like characteristics (Mussa-Ivaldi et al., 1985; Artemiadis et al., 2010). As the estimation interval in this work is significantly shorter than the ones in Mussa-Ivaldi et al. (1985) and Artemiadis et al. (2010), we assume that the stiffness ${\bar{K}}_{q}$ is symmetric for *t*_p_ ∈ [0, *T*_est_]. As the potential energy of the vector field of the involuntary feedback forces must increase in response to deviations from the unperturbed states, the symmetric stiffness ${\bar{K}}_{q}$ must also be positive definite. In order to incorporate both symmetry and positive definiteness of the stiffness ${\bar{K}}_{q}$ in the estimation, we apply the Cholesky decomposition ${\bar{K}}_{q}={\bar{L}}_{q}{\bar{L}}_{q}^{\text{T}}$, where ${\bar{L}}_{q}$ is a lower triangular matrix (Horn and Johnson, 2012). With this decomposition and the a priori determined values of the estimated BIP vector $\hat{\bar{\pi }}$, the unknown model parameter vector ***ζ*** reduces to

(36) $$\begin{array}{l} \bar{\zeta }={\left[{\bar{D}}_{q,11},{\bar{D}}_{q,12},{\bar{D}}_{q,21},{\bar{D}}_{q,22},{\bar{L}}_{q,11},{\bar{L}}_{q,12},{\bar{L}}_{q,22}\right]}^{\text{T}} \end{array}$$

and the elements of the output vector ***b*** are replaced by

(37) $$\begin{array}{l} \bar{b}=\Delta {\hat{\tau }}_{\text{ext}}-\Delta \bar{Y}\left(q,{\hat{q}}^{*},\dot{q},{\dot{\hat{q}}}^{*},\ddot{q},{\ddot{\hat{q}}}^{*}\right)\hat{\bar{\pi }}. \end{array}$$

Due to the non-linearity introduced by ${\bar{K}}_{q}={\bar{L}}_{q}{\bar{L}}_{q}^{\text{T}}$, we can no longer use the identification model in (31). Instead, we apply non-linear least squares analysis to minimize ${\left\|f(\bar{\zeta })\right\|}^{2}$ with

(38) $$\begin{array}{l} f\left(\bar{\zeta }\right)={\bar{D}}_{q}\Delta \dot{\hat{q}}+{\bar{L}}_{q}{\bar{L}}_{q}^{\text{T}}\Delta \hat{q}-\bar{b}\;. \end{array}$$

to determine the estimated model parameter vector $\hat{\bar{\zeta }}$, i.e., estimated damping ${\hat{\bar{D}}}_{q}$ and stiffness ${\hat{\bar{K}}}_{q}$.

### 2.3. Simulation

We validate our method with simulated data of a neuromechanical model of the human arm, which generates transversal movements of a two-link, six-muscle arm through calculation of muscle activities (Franklin et al., 2008). By simulating each movement twice, once with and once without perturbation, we validate the estimation of the unperturbed dynamics. Furthermore, we use the inertial characteristics and the intrinsic impedance to validate the estimation of the involuntary impedance parameters.

We use the neuromechanical model to simulate a static postural task and a dynamic movement task. As the static postural task does not include an underlying movement, we are able to apply the do-not-intervene-voluntarily paradigm. While the do-not-intervene-voluntarily paradigm does not guarantee exclusion of voluntary feedback or constant unperturbed states (Sainburg, 2015), it is widely used and accepted as a plausible approximation of both of these assumptions (Mussa-Ivaldi et al., 1985; Gomi and Kawato, 1997; Osu and Gomi, 1999). Thus, given the absence of an underlying movement, and the fact that the elements of the BIP vector $\bar{\pi }$ are all constant, we are able to use an estimation interval duration *T*_est_ that is longer than the delay of voluntary feedback δ_v_ in the static postural task. As the dynamic movement task does include an underlying movement, it is performed without the do-not-intervene-voluntarily paradigm and we use the limited estimation interval duration *T*_est_ = δ_v_.

#### 2.3.1. Human Arm Model

The rigid body dynamics of the neuromechanical model are determined by (1) and the correlations between the internal torques ***τ***_int_ and the muscle tensions ***m*** are determined by (2). The muscle tensions

(39) $$\begin{array}{l} m=m_{a}+m_{\text{imp}}\;, \end{array}$$

where ***m***_*a*_ and ***m***_imp_ represent the muscle tensions due to muscle activation and mechanical impedance, respectively. The muscle tensions due to muscle activation ***m***_*a*_ are assumed to be identical to the muscle activations ***a*** in (3) and the muscle tensions due to mechanical impedance

(40) $$\begin{array}{l} m_{\text{imp}}=D_{m}{\dot{e}}_{m}+K_{m}e_{m} \end{array}$$

(41) $$\begin{array}{l} D_{m}=K_{m}/12\;,\;K_{m}=K_{0}+K_{1}a\;, \end{array}$$

where *D*_*m*_, *K*_*m*_, and ***e***_*m*_ represent damping, stiffness, and tracking errors with respect to the desired trajectory at the muscle level and *K*_0_ and *K*_1_ contain intrinsic stiffness parameters. The feedforward muscle activations ***a***_FF_ are calculated a priori based on the inverse kinematics and dynamics of the desired movement. The feedback muscle activations ***a***_FB_ are modeled by proportional-derivative (PD) control that depends on the tracking errors ***e***_*m*_, the respective derivatives ${\dot{e}}_{m}$, and a simulated feedback delay δ_s_ = 60 ms. The signal-dependent noise ***a***_N_ is calculated with zero mean Brownian motion, which provides movements similar to those observed in previous experiments (Burdet et al., 2001). It is generated by a normally distributed random variable, which is amplified by a parameter α_N_ = 12.5 and filtered by a fifth order low pass Butterworth filter with a cut-off frequency *f*_c,N_ = 2 Hz.

In the implementation of the neuromechanical model, there is no distinction between reflexive and voluntary feedback. Instead, both feedback mechanisms are combined and the simulated feedback delay δ_s_ is defined to lie between the respective delays δ_r_ and δ_v_. As the simulated feedback delay δ_s_ is shorter than the duration of our estimation interval *T*_est_, the feedback behavior within the estimation interval is influenced by voluntary contributions. In order to avoid this and enable the validation of the estimated involuntary impedance parameters ${\hat{\bar{D}}}_{q}$ and ${\hat{\bar{K}}}_{q}$ through the simulated intrinsic impedance components, we use a variation, in which the simulated feedback delay δ_s_ is defined equal to the delay δ_v_ of voluntary feedback, i.e., δ_s_ = δ_v_ = 115 ms. Although the physiological correctness of the variation is slightly reduced compared to the original implementation, it nonetheless represents a plausible simulation of human behavior. More importantly, it represents a means for validation of our method and for comparison of its performance to those of existing methods. In order to represent the effects of the do-not-intervene-voluntarily paradigm, voluntary feedback is removed in the simulations of the static postural task.

#### 2.3.2. Simulation Design

As the definition of the signal-dependent noise ***a***_N_ is based on a normally distributed random variable, the static task and the dynamic task are each simulated ten times and the respective results are averaged. In both tasks, the simulated manipulandum inertia and damping are defined according to the admittance inertia *M*_adm_ = diag{5, 5} kg and damping *D*_adm_ = diag{20, 20} Ns/m of our apparatus.

**Figure 5:**
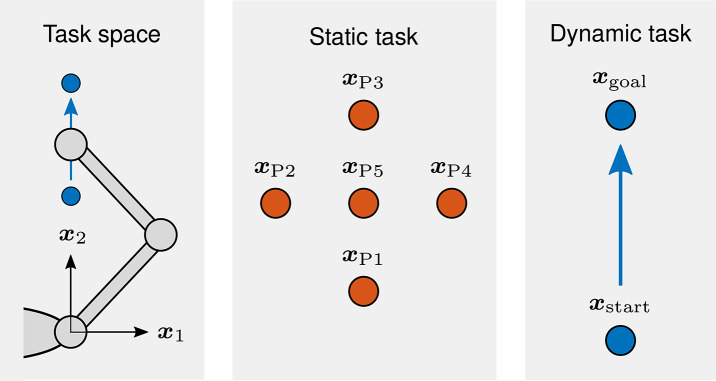
Schematic representation of the human arm task space, the static task, and the dynamic task. In the static task, the arm maintains a total of five different positions that are distributed evenly across the horizontal plane. In the dynamic task, it performs point to point movements along the sagittal axis.

***Static task***. In the static task, the arm maintains a total of five different positions that are distributed evenly across the horizontal plane: $x_{\text{P}1}={\left[0,0.35\right]}^{\text{T}}\text{ m}$, $x_{\text{P}2}={\left[-10,0.45\right]}^{\text{T}}\text{ m}$, $x_{\text{P}3}={\left[0,0.55\right]}^{\text{T}}\text{ m}$, $x_{\text{P}4}={\left[10,0.45\right]}^{\text{T}}\text{ m}$, and $x_{\text{P}5}={\left[0,0.45\right]}^{\text{T}}\text{ m}$ (see Figure 5). In order to obtain perturbations that are similar to those of the static estimations in Gomi and Kawato (1997), we define the perturbation profile duration *T*_p,1_ = 160 ms which results in a total perturbation duration *T*_pert_ = 400 ms. We change the interaction mode from closed-loop to open-loop which means that applied forces do not contribute to the movement of the manipulandum. As this means that we are essentially applying a position perturbation, we are able to define the amplitude of the perturbation position profile *x*_pert_, which we set to 8 mm. The perturbation angles ϕ_pert_ are defined by the set Φ_pert_ = {(π/12)*k* | *k* = 1 − 24 \ {6, 12, 18, 24}}, which contains 20 angles. Each perturbation angle ϕ_pert_ is executed once in every position and the order of the resulting perturbations is randomized. Single execution of each of the 20 perturbation angles ϕ_pert_ in each of the five positions results in a total of 100 trials. The duration of the estimation interval *T*_est_ = 400 ms and the estimated BIP vector $\hat{\bar{\pi }}$ is obtained by calculating the least squares solution for the complete data set of all 100 trials.

***Dynamic task***. In the dynamic task, the arm performs two-dimensional point to point movements along the sagittal axis from $x_{\text{start}}={\left[0,0.30\right]}^{\text{T}}\text{ m}$ to $x_{\text{goal}}={\left[0,0.55\right]}^{\text{T}}\text{ m}$ (see Figure 5). The duration of the movements *T*_mov_ is set to 2 s. The feedforward muscle activations ***a***_FF_ are calculated with the inverse kinematics and dynamics of a positional data set, which is provided by the authors of Franklin et al. (2008). It contains positional data of 50 two-dimensional point to point arm movements, which possess identical start and goal positions as the movements in our task. The perturbations are designed to generate sufficiently large deviations in as short a time frame as possible, but nonetheless be physically plausible and kinesthetically renderable under hardware limitations. The perturbation amplitude *A*_pert_ = 40 N and the perturbation profile duration *T*_p,1_ = 70 ms which corresponds to a total perturbation duration *T*_pert_ = 175 ms. The perturbation is initiated when the hand reaches *x*_2_ = 0.4 m which equals a distance of 0.1 m along the axis of the principal movement. The perturbation angles ϕ_pert_ are defined by Φ_pert_, which is identical to the static task. Each perturbation angle ϕ_pert_ is executed three times and the order of the resulting perturbations is randomized. Three repetitions of each of the 20 perturbation angles ϕ_pert_ result in a total of 60 trials, of which each consists of an unperturbed and a perturbed movement. The duration of the estimation interval *T*_est_ = 115 ms and the estimated damping ${\hat{\bar{D}}}_{q}$ and stiffness ${\hat{\bar{K}}}_{q}$ are obtained by calculating the least squares solutions for data sets of 20 trials each. Grouping the complete data set of all 60 trials into data sets of 20 trials each, starting from the first 20 trials and moving the respective data set along trial by trial until the last 20 trials, provides a total of 41 individual least squares solutions.

### 2.4. Experiment

In order to evaluate the performance of the proposed method for real data, we conduct an experiment with 12 participants. The experiment design is identical to the simulation design, apart from a randomized time interval before the onset of the perturbation in the static task and a randomized distribution of unperturbed and perturbed trials in the dynamic task.

#### 2.4.1. Apparatus and Experiment Design

**Figure 6:**
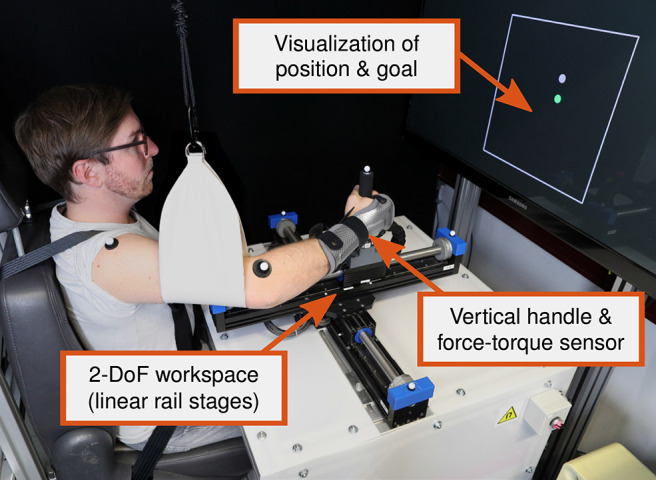
Reenactment of an individual interacting with the apparatus (during the dynamic task of the experiment). Informed written consent for the publication of this image was obtained from the individual.

The apparatus is presented in Figure 6. It consists of two linear servo motor driven single rail stages (*Copley Controls Thrusttube Module*) that are mounted on top of each other in orthogonal orientation. Together, they span a 2-DoF workspace of ±0.20 m and are each equipped with an encoder that yields position data with a precision of 1 μm. Additionally, six motion capture cameras (*Qualisys Miqus*) are available for tracking of passive markers. A vertical handle and a 6-DoF force-torque sensor (*JR3-67M25*) are mounted on top of the upper servo motor driven cart to measure the forces in the horizontal plane. A custom-built seat with shoulder belts, a sling attached to the ceiling, and a wrist orthosis are available for limitation of the movements of the participants. Visual feedback is provided on a screen behind the apparatus and implemented with the Psychophysics Toolbox (Brainard, 1997). The position of the cart is visualized by a dot and the workspace safety boundaries are visualized in the form of a boundary box.

Haptic interaction by means of participant force input is enabled by the admittance control scheme defined in (26), where *M*_adm_ = diag{5, 5} kg and *D*_adm_ = diag{20, 20} Ns/m. The parameters of the admittance control scheme are chosen to guarantee natural interaction with the apparatus and sufficient attenuation of force-torque sensor noise. Precise rendering of the position is ensured by a high gain PD controller, which is implemented in *Matlab/Simulink* and executed on a *Linux* system with *RT-preempt* real-time kernel (*Ubuntu 14.04, 3.14.3-rt4-prt*). The sample rate is set to *f*_*s*_ = 4 kHz and inputs to the Thrusttube Modules are downsampled to 2 kHz due to hardware limitations. The signals are filtered using a seventh order Savitzky-Golay polynomial filter with a cut-off frequency of *f*_c,SG_ = 50 Hz.

In order to avoid predictability of the perturbations and adaptation of the participants, a randomized time interval with *T*_random_ ∈ [1, 3] s is included before the onset of the perturbations in the static task. For the same reasons and to obtain both unperturbed and perturbed movements, the ratio of perturbed trials to total amount of trials is set to 33 % in the dynamic task. In order to avoid effects of fatigue, the resulting 180 trials of the dynamic task are performed in three consecutive blocks. Each of these blocks contains a total of 60 trials that consist of 20 perturbed and 40 unperturbed randomly distributed trials. In both tasks, the visual feedback of the position of the cart is deactivated during the perturbations.

#### 2.4.2. Participants and Experiment Procedure

A total of 12 participants (9 males, 3 females) with between-subject mean (SD) age of 26.92 (3.40) years, height of 174.83 (7.38) cm, and weight of 69.43 (9.82) kg volunteered to participate in this experiment. All 12 participants had normal or corrected-to-normal vision. One of the participants was left handed and performed the experiment with the non-dominant hand. The remaining 11 participants were right handed and participated with the dominant hand. Informed written consent was obtained from all participants before the experiment, which was conducted according to the principles in the Declaration of Helsinki. The research ethics were obtained from the ethics committee at the Technical University of Munich.

The participants were seated in front of the apparatus and their upper body was restrained by two shoulder belts to fix the position of the shoulder. Their upper arm was supported by a sling attached to the ceiling to constrain all motions to the horizontal plane and reduce effects of fatigue. In order to avoid wrist motions, the participants were wearing a wrist orthosis. Two passive motion capture markers were placed on shoulder and elbow to measure the lengths of the upper arm and the forearm.

In the beginning of the static task, the cart automatically moved to the first position at the bottom of the workspace. After the application of all 20 perturbations, it automatically moved to the next position. This procedure was repeated until all 100 perturbations were completed. Analogous to the simulation, each perturbation angle ϕ_pert_ was executed once in each of the positions and the order was randomized. The participants were informed about the procedure, including the perturbations and the randomized time interval before the onset of the perturbation. They were told that their objective was to naturally grasp the handle on top of the cart. Additionally, they were instructed to not voluntarily react to the perturbations in any way and not prepare for them in any kind of preemptive manner, e.g., by co-contraction.

In the beginning of the dynamic task, the cart automatically moved to the start position at the bottom of the workspace. As soon as it reached the start position, the admittance turned on. As soon as the participants reached the goal position, represented by a dot at the top of the workspace, the admittance turned off and the cart automatically moved back down to the start position. This procedure was repeated until all 60 trials with 20 randomly distributed perturbed trials were completed. In total, three of these blocks of trials were completed for a total of 180 trials. Analogous to the simulation, each perturbation angle ϕ_pert_ was executed three times and the order was randomized. The participants were informed about the procedure, including the perturbations and their random distribution. They were told that their objective was to naturally grasp the handle on top of the cart and move it from the start position to the goal position. They were told that the duration of the movement should be ~ 2 s. In order to help them adjust their movement velocity, a beep sound occurred after 2 s. The participants were informed that they were allowed to voluntarily react to the perturbations. Additionally, they were instructed to not prepare for the perturbations in any kind of preemptive manner, e.g., by co-contraction.

## 3. Results

In this section, we first present the results of the simulation, which consist of the validation of the feedback jerk isolation and the involuntary impedance estimation. Subsequently, we present the results of the evaluation with experimental data. The results of the simulation are either presented in within-, between-, or cross-simulation mean (SD). The cross-simulation mean (SD) is obtained by calculating the mean of the within-simulation means and the mean of the within-simulation SDs. Analogous correlations apply for the results of the experiment, which are presented in within-, between-, or cross-subject mean (SD). As the majority of the results are cross-simulation/cross-subject mean (SD) results, for simplicity, from this point on, the respective results are referred to simply as mean (SD) results.

### 3.1. Validation Feedback Jerk Isolation

In this section, we validate the feedback jerk isolation by analyzing its performance for different movement velocities and variations of neural noise. Furthermore, we compare the results to those obtained by application of the methods in Gomi and Kawato (1997) and Erden and Billard (2015). In order to ensure equal conditions and enable performance comparisons without effects of voluntary feedback, all methodologies are applied to the same sets of simulated data and use the same duration of the estimation interval *T*_est_ = 115 ms. The estimation of the unperturbed dynamics $\left\{q^{*},{\dot{q}}^{*},{\ddot{q}}^{*},\tau_{\text{ext}}^{*}\right\}$ is validated by analysis of the estimated variational dynamics $\left\{\Delta \hat{x},\Delta \dot{\hat{x}},\Delta \ddot{\hat{x}},\Delta {\hat{u}}_{\text{ext}}\right\}$. The estimation accuracy is assessed using the normalized root mean square errors (NRMSEs) in the estimation interval [0, *T*_est_]:

(42) $$\begin{array}{l} \text{NRMSE}=\sqrt{\frac{1}{n}\overset{n}{\underset{i=1}{\sum }}{\left(\frac{\chi_{i}-{\hat{\chi }}_{i}}{\nu_{i}}\right)}^{2}}\;, \end{array}$$

where ν is the normalizing value, χ is the real value, $\hat{\chi }$ is the estimated value, and *n* = 2 is the dimensionality. For the estimated variational dynamics $\left\{\Delta \hat{x},\Delta \dot{\hat{x}},\Delta \ddot{\hat{x}},\Delta {\hat{u}}_{\text{ext}}\right\}$, the normalizing value ν is given by the maximum real value in the estimation interval [0, *T*_est_]. In order to analyze the performance for different movement velocities, the duration of the movements *T*_mov_ is changed (1, 3 s). For analysis of the performance for different variations of neural noise, the parameterization of the zero mean Brownian motion is changed in terms of the cut-off frequency *f*_c,N_ (1, 3 Hz) and the amplitude α_N_ (5, 20).

#### 3.1.1. Filter Configuration

**Figure 7:**
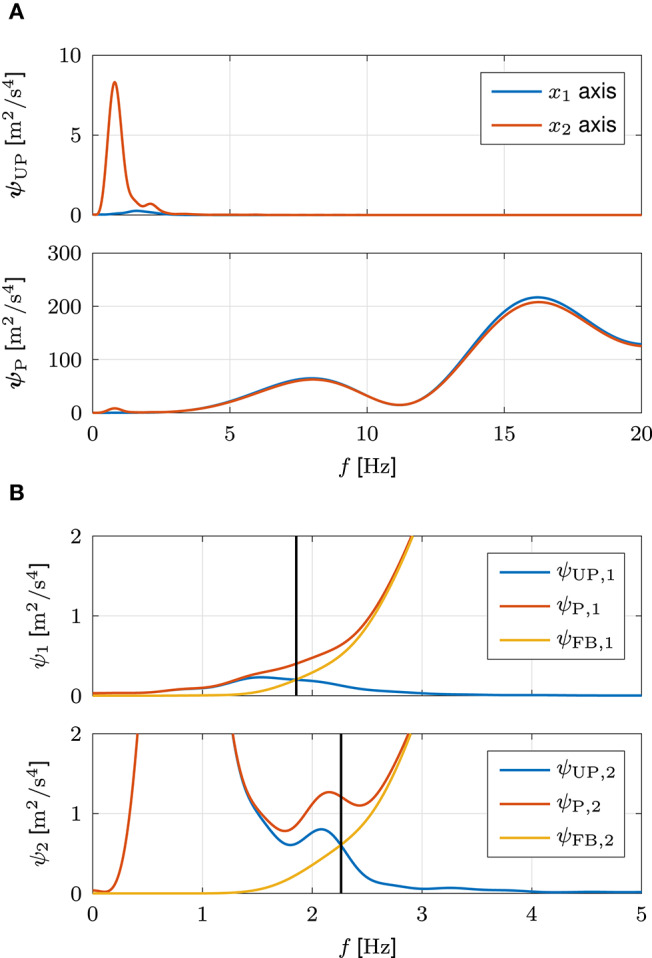
Filter configuration based on simulated data. **(A)** Mean results of the ESDs ***ψ***_UP_ and ***ψ***_P_ of the jerks $\overset{⃛}{x}$ of the unperturbed and perturbed movements. **(B)** Calculation of the cut-off frequencies ***f***_c,HP_, defined as the frequencies ***f*** at which the ESDs ***ψ***_FB_ of the jerks $\overset{⃛}{x}$ of the evoked feedback behavior become larger than the ESDs ***ψ***_UP_ of the jerks $\overset{⃛}{x}$ of the unperturbed movements. For clarity, the calculation is exemplarily illustrated for a single simulation and both graphs are limited to the low frequency ranges. The respective cut-off frequencies ***f***_c,HP_ are indicated by vertical lines.

Figure 7A shows the mean results of the ESDs ***ψ***_UP_ and ***ψ***_P_. In the unperturbed movements, the energy of the principal movement along the *x*_2_ axis is distinguishable by a peak in the respective ESD ψ_UP,2_. A secondary, much lower peak represents effects of neural noise. As the point to point movements do not require movements along the *x*_1_ axis, the respective ESD ψ_UP,1_ is significantly lower and represents effects of neural noise. The energy of both axes of the unperturbed movements decreases to marginally low values for high frequencies. The opposite applies for the energy of the perturbed movements, which increases to significantly higher values for high frequencies. The respective ESDs ***ψ***_P_ are much higher than those of the unperturbed movements and have multiple peaks in the high frequency range.

Figure 7B shows the ESDs ***ψ***_UP_, ***ψ***_P_, and ***ψ***_FB_ of a single simulation in two axis-specific graphs to demonstrate the calculation of the cut-off frequencies ***f***_c,HP_. The cut-off frequency *f*_c,HP,2_ of the *x*_2_ axis is sufficiently high to lie above the energy of the principal movement. Concurrently, it is sufficiently low to lie below the energy of the feedback behavior. Due to the absence of movements along the *x*_1_ axis, the cut-off frequency *f*_c,HP,1_ is slightly lower. The between-simulation mean (SD) results of the cut-off frequencies ***f***_c,HP_ of all ten simulations are ***f***_c,HP_ = [1.89 (0.08), 2.24 (0.04)] Hz.

#### 3.1.2. Results

**Figure 8:**
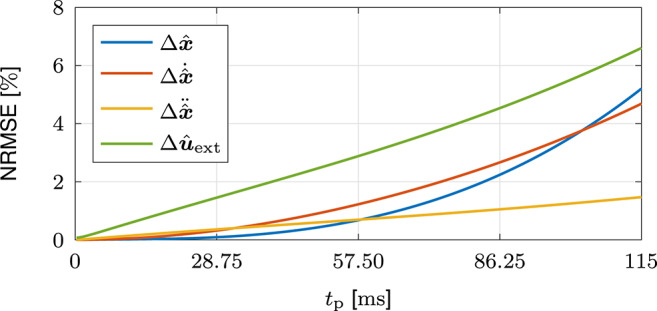
Validation of feedback jerk isolation with simulated data. Mean results of the NRMSEs of the estimated variational dynamics $\left\{\Delta \hat{x},\Delta \dot{\hat{x}},\Delta \ddot{\hat{x}},\Delta {\hat{u}}_{\text{ext}}\right\}$ in the estimation interval [0, *T*_est_].

The mean results of the NRMSEs in Figure 8 show that the NRMSEs all increase with *t*_p_. The gradual increase of the NRMSEs of the estimated variational accelerations $\Delta \ddot{\hat{x}}$ results from the integration of the estimated variational jerks $\Delta \overset{⃛}{\hat{x}}$, which include estimation errors that originate from the calculation of the high pass filtered jerks ${\overset{⃛}{x}}_{\text{HP}}$, i.e., the separation of the unperturbed and the variational behavior. The increases of the NRMSEs of the estimated variational velocities $\Delta \dot{\hat{x}}$ and positions $\Delta \hat{x}$ result from the consecutive integrations of the estimated variational accelerations $\Delta \ddot{\hat{x}}$. The slightly larger NRMSEs of the estimated variational external forces $\Delta {\hat{u}}_{\text{ext}}$ are caused by the estimation errors of the estimated unperturbed velocities ${\dot{\hat{x}}}^{*}$ and accelerations ${\ddot{\hat{x}}}^{*}$. Despite the increase of the NRMSEs with *t*_p_, the maximum values of the estimated variational kinematics $\left\{\Delta \hat{x},\Delta \dot{\hat{x}},\Delta \ddot{\hat{x}}\right\}$ are all below 6 % and the NRMSE of the estimated variational external forces $\Delta {\hat{u}}_{\text{ext}}$ is below 8 %.

#### 3.1.3. Comparison

**Figure 9:**
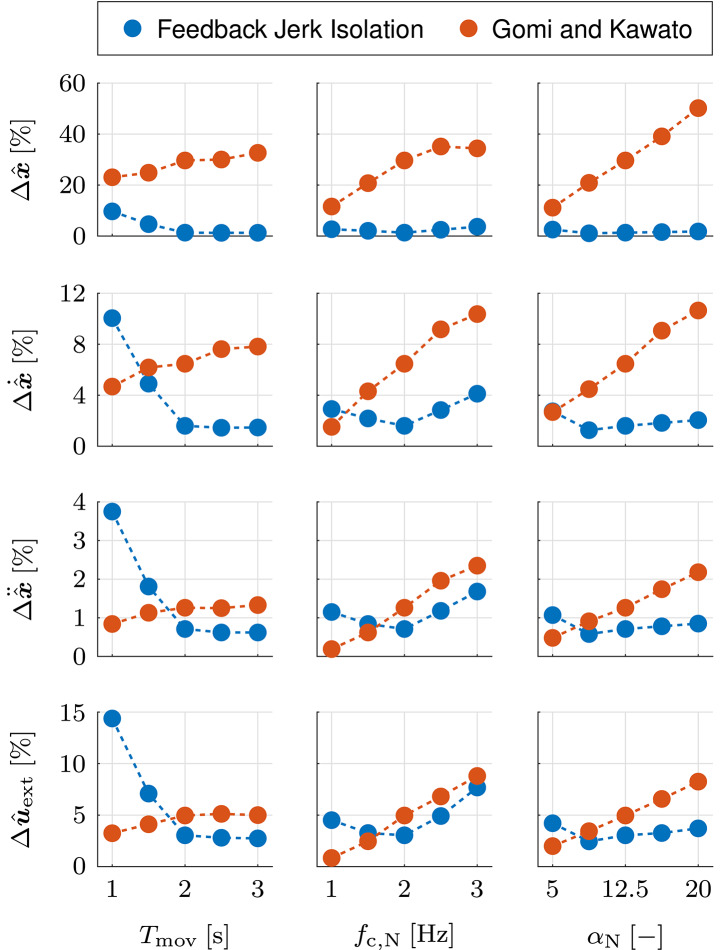
Validation of feedback jerk isolation with simulated data. Mean results of the NRMSEs of the estimated variational dynamics $\left\{\Delta \hat{x},\Delta \dot{\hat{x}},\Delta \ddot{\hat{x}},\Delta {\hat{u}}_{\text{ext}}\right\}$, averaged for the estimation interval [0, *T*_est_], over different movement durations *T*_mov_, cut-off frequencies *f*_c,N_, and amplitudes α_N_.

In order to compare the results to those obtained with the methods in Gomi and Kawato (1997) and Erden and Billard (2015), we average the NRMSEs over the complete estimation interval [0, *T*_est_]. For simplicity, from this point on, we refer to the resulting averaged NRMSEs simply as NRMSEs. The mean (SD) results of the NRMSEs for the original simulation are listed in Table 1A and those for different durations of the movements *T*_mov_, cut-off frequencies *f*_c,N_, and amplitudes α_N_ are listed in Tables 1B–D, respectively. The abbreviations FJI, GOM, and ERD represent our feedback jerk isolation and the methods in Gomi and Kawato (1997) and Erden and Billard (2015), respectively. In the discussion of the NRMSEs of the alternative simulations, the changes in configurations and results are evaluated relative to the original simulation. In order to facilitate the comparisons of the NRMSEs of the original and alternative simulations, Tables 1B–D also include the NRMSEs of the original simulation, and the contents of all three tables are additionally illustrated with intermediate values in Figure 9.

**Table 1:** Validation of feedback jerk isolation with simulated data.

**(A) ORIGINAL CONFIGURATION**				
**Method**	**$\Delta \hat{x}[\%]$**	**$\Delta \dot{\hat{x}}[\%]$**	**$\Delta \ddot{\hat{x}}[\%]$**	**$\Delta {\hat{u}}_{\text{ext}}[\%]$**
FJI	1.33 (1.16)	1.60 (1.42)	0.71 (0.62)	3.04 (2.91)
GOM	29.65 (27.04)	6.47 (6.04)	1.26 (1.18)	4.96 (4.97)
ERD	911.10 (750.51)	551.24 (468.13)	5.31 (3.96)	17.77 (15.03)

**Table:** 

**(B) MOVEMENT DURATION** ***T*_mov_**					
**Method**	***T*_mov_ [s]**	**$\Delta \hat{x}[\%]$**	**$\Delta \dot{\hat{x}}[\%]$**	**$\Delta \ddot{\hat{x}}[\%]$**	**$\Delta {\hat{u}}_{\text{ext}}[\%]$**
FJI	1.0	9.67 (6.50)	10.05 (6.56)	3.75 (2.30)	14.38 (10.12)
FJI	2.0	1.33 (1.16)	1.60 (1.42)	0.71 (0.62)	3.04 (2.91)
FJI	3.0	1.29 (1.11)	1.47 (1.32)	0.62 (0.57)	2.74 (3.04)
GOM	1.0	23.06 (20.53)	4.68 (3.93)	0.84 (0.77)	3.24 (3.31)
GOM	2.0	29.65 (27.04)	6.47 (6.04)	1.26 (1.18)	4.96 (4.97)
GOM	3.0	32.64 (29.79)	7.82 (7.36)	1.33 (1.29)	5.00 (5.24)
**(C) CUT-OFF FREQUENCY** ***f*_c,N_**					
**Method**	***f*_c,N_ [Hz]**	**$\Delta \hat{x}[\%]$**	**$\Delta \dot{\hat{x}}[\%]$**	**$\Delta \ddot{\hat{x}}[\%]$**	**$\Delta {\hat{u}}_{\text{ext}}[\%]$**
FJI	1.0	2.67 (2.17)	2.92 (2.42)	1.15 (0.98)	4.51 (3.79)
FJI	2.0	1.33 (1.16)	1.60 (1.42)	0.71 (0.62)	3.04 (2.91)
FJI	3.0	3.66 (2.75)	4.12 (3.22)	1.68 (1.35)	7.68 (7.42)
GOM	1.0	11.58 (10.30)	1.52 (1.41)	0.19 (0.16)	0.84 (0.81)
GOM	2.0	29.65 (27.04)	6.47 (6.04)	1.26 (1.18)	4.96 (4.97)
GOM	3.0	34.39 (32.82)	10.37 (8.73)	2.35 (2.21)	8.80 (8.75)
**(D) AMPLITUDE** **α_N_**					
**Method**	**α_N_ [−]**	**$\Delta \hat{x}[\%]$**	**$\Delta \dot{\hat{x}}[\%]$**	**$\Delta \ddot{\hat{x}}[\%]$**	**$\Delta {\hat{u}}_{\text{ext}}[\%]$**
FJI	5.0	2.54 (2.09)	2.74 (2.32)	1.07 (0.93)	4.21 (3.59)
FJI	12.5	1.33 (1.16)	1.60 (1.42)	0.71 (0.62)	3.04 (2.91)
FJI	20.0	1.79 (1.50)	2.05 (1.79)	0.85 (0.75)	3.71 (4.03)
GOM	5.0	11.14 (9.73)	2.68 (2.73)	0.48 (0.40)	1.99 (1.99)
GOM	12.5	29.65 (27.04)	6.47 (6.04)	1.26 (1.18)	4.96 (4.97)
GOM	20.0	50.18 (49.74)	10.65 (9.51)	2.18 (2.11)	8.25 (8.33)

*Mean (SD) results of the NRMSEs of the estimated variational dynamics $\left\{\Delta \hat{x},\Delta \dot{\hat{x}},\Delta \ddot{\hat{x}},\Delta {\hat{u}}_{\text{ext}}\right\}$ averaged for the estimation interval [0, T_est_]. **(A)** Original configuration. **(B)** Different movement durations T_mov_. **(C)** Different cut-off frequencies f_c,N_. **(D)** Different amplitudes α_N_. The abbreviations FJI, GOM, and ERD represent our feedback jerk isolation and the methods in Gomi and Kawato (1997) and Erden and Billard (2015), respectively*.

The NRMSEs in Table 1A show that FJI achieves high estimation accuracy and outperforms both GOM and ERD. The magnitudes and differences of the individual NRMSEs of FJI are in accordance with those of the NRMSEs presented in Figure 8. The performance difference of FJI and GOM increases from a small difference in $\Delta \ddot{\hat{x}}$ to a large difference in $\Delta \hat{x}$ and the performance difference in $\Delta {\hat{u}}_{\text{ext}}$ is marginally larger than that of $\Delta \ddot{\hat{x}}$. These correlations are plausible, due to the dependency on the averaged unbiased dynamics of all perturbed movements. While forces and accelerations are of similar magnitude, velocities and especially positions vary between movements. As a consequence, the accuracy of the averaged unbiased dynamics decreases significantly from accelerations down to positions. Similar correlations apply to the performance difference of FJI and ERD, but with significantly larger differences, especially for $\Delta \dot{\hat{x}}$ and $\Delta \hat{x}$. The magnitudes of the NRMSEs of ERD are plausible, due to the dependency on the constant values of the offsets at perturbation onset. While these constant values represent accurate approximations of the unperturbed states of the quasi-static movements of the manual welding task in Erden and Billard (2015), they are incapable of accurately representing the kinematics of the unperturbed states of dynamic point to point movements. For this reason, we exclude this method from the remainder of the validation of the feedback jerk isolation and the involuntary impedance estimation.

The NRMSEs in Table 1B show that the performance of FJI is decreased for a higher movement velocity (resulting from a shorter duration of the movement) and marginally increased for a lower movement velocity (resulting from a longer duration of the movement). A higher movement velocity results in an increased cut-off frequency of the high pass filter which causes an increased information loss in the isolation of the feedback jerk. As a result, the estimation accuracy is decreased. The marginal increase in performance for a lower movement velocity suggests that the corresponding NRMSEs are close to the smallest possible errors, which are caused by unavoidable frequency overlap of unperturbed movements and feedback behavior, neural noise, and imperfect properties of a non-ideal high pass filter. The performance of GOM is slightly increased for a higher movement velocity and slightly decreased for a lower movement velocity. As the accumulated influence of the neural noise at the onset of the perturbation is negatively correlated with the movement velocity, the deviations from the averaged unbiased dynamics are larger for slow movements than they are for fast movements. As the performance of GOM is directly influenced by these deviations, the estimation accuracy is positively correlated with the different movement velocities. For *T*_mov_ = 1 s, GOM outperforms FJI in all NRMSEs except $\Delta \hat{x}$, for which the performance difference is decreased compared to Table 1A. For *T*_mov_ = 3 s, FJI outperforms GOM with slightly increased performance differences compared to Table 1A.

The NRMSEs in Table 1C show that the performance of FJI is slightly decreased for lower and higher cut-off frequencies of the noise, with the latter resulting in slightly larger performance differences than the former. It seems that the lower cut-off frequency of the noise results in such a decreased cut-off frequency of the high pass filter, that it is too close to the frequency content of the unperturbed behavior. For the higher cut-off frequency of the noise, the increased cut-off frequency of the high pass filter results in an increased information loss in the isolation of the feedback jerk. Despite these slight decreases in performance, FJI still achieves high estimation accuracy for both alternative simulations. The performance of GOM is increased/decreased for lower/higher cut-off frequencies of the noise due to smaller/larger deviations from the averaged unbiased dynamics. For *f*_c,N_ = 1 Hz, GOM outperforms FJI in all NRMSEs except $\Delta \hat{x}$, for which the performance difference is decreased compared to Table 1A. For *f*_c,N_ = 3 Hz, FJI outperforms GOM with slightly increased performance differences compared to Table 1A.

The NRMSEs in Table 1D illustrate that a lower amplitude of the noise has an almost identical effect on the performance as a lower cut-off frequency of the noise in Table 1C. In contrast, a higher amplitude of the noise has very different effects. For FJI, a higher amplitude of the noise only leads to a marginal decrease of the performance, much smaller than that in Table 1C. For GOM, it leads to a similar decrease of the performance as in Table 1C, except for $\Delta \hat{x}$, for which the decrease is much larger. For α_N_ = 5, the performance difference of FJI and GOM is almost identical to that of *f*_c,N_ = 1 Hz in Table 1C. For α_N_ = 20, FJI outperforms GOM with increased performance differences compared to Table 1A, especially for $\Delta \hat{x}$. The different effects emphasize three issues: (1) For GOM, the estimation accuracy strongly depends on the similarity to the averaged unbiased dynamics. (2) For FJI, it depends more strongly on the frequency of the noise than it does on the amplitude, due to the influence on the cut-off frequency of the high pass filter. (3) The performance difference in GOM due to a higher amplitude of the noise is larger than the performance difference in FJI due to a higher frequency of the noise, especially for $\Delta \hat{x}$.

### 3.2. Validation Involuntary Impedance Estimation

In this section, we validate and compare the performance of the involuntary impedance estimation. The estimation accuracy is assessed using the normalized absolute errors (NAEs) of the estimated values. The normalizing values are either given by the real values of the elements of the BIP vector $\bar{\pi }$ or the maximum real values of the elements of the damping ${\bar{D}}_{q}$ and stiffness ${\bar{K}}_{q}$, which are obtained by averaging the respective elements for the estimation interval [0, *T*_est_]. For comparability of the results, we transform the inertial parameters in Gomi and Kawato (1997) to the elements of the BIP vector $\bar{\pi }$.

In order to obtain additional means of comparing the overall performance of the methods, we calculate two additional performance criteria that are based on the Akaike information criterion (AIC) and the Bayesian information criterion (BIC). When comparing least squares fitted models, the AIC and the BIC are defined by the residual sum of squares (RSS) of the real values and the estimated model outputs:

(43) $$\begin{array}{l} \text{AIC}=2p+k\ln\left(\text{RSS}\right)\;, \end{array}$$

(44) $$\begin{array}{l} \text{BIC}=\ln\left(k\right)p+k\ln\left(\text{RSS}\right)\;, \end{array}$$

(45) $$\begin{array}{l} \text{RSS}=\frac{1}{n}\overset{n}{\underset{i=1}{\sum }}(\overset{k}{\underset{j=1}{\sum }}{\left(y_{i,j}-{\hat{y}}_{i,j}\right)}^{2})\;, \end{array}$$

where *y* is the real value, *ŷ* is the estimated model output, *p* is the number of parameters, *k* is the number of data points, and *n* = 2 is the dimensionality (Burnham and Anderson, 2002). As we aim to compare the overall performance of the methods, we define the estimated model output ŷ to be the simulation output that is obtained by inserting the estimated BIP vector $\hat{\bar{\pi }}$, damping ${\hat{\bar{D}}}_{q}$, and stiffness ${\hat{\bar{K}}}_{q}$ into the neuromechanical model of the human arm (within the estimation interval [0, *T*_est_]), and the real value *y* to be the corresponding simulation output of the original simulation.

#### 3.2.1. Results

Table 2A contains the mean (SD) results of the NAEs of the estimated BIP vector $\hat{\bar{\pi }}$, damping ${\hat{\bar{D}}}_{q}$, and stiffness ${\hat{\bar{K}}}_{q}$. The NAEs of the elements of the estimated BIP vector $\hat{\bar{\pi }}$ do not possess SDs, because they are estimated with the complete data set of the static task. The NAEs of all three elements are below 2% which indicates high estimation accuracy of the combination of the dynamic regressor representation with the data of the static task. The NAEs of the elements of the estimated damping ${\hat{\bar{D}}}_{q}$ and stiffness ${\hat{\bar{K}}}_{q}$ are all approximately equal to or below 10% and demonstrate high estimation accuracy of the non-linear least squares estimation with the data of the dynamic task. The NAEs of the elements ${\hat{\bar{D}}}_{q,11}$ and ${\hat{\bar{K}}}_{q,11}$ are slightly increased compared to the remaining elements of the respective matrices. This slight increase is plausible, as these elements represent the contributions of the single-joint shoulder muscles, which are less involved due to less movement of the shoulder joint.

**Table 2:** Validation of involuntary impedance estimation with simulated data.

**(A) NAES OF THE ELEMENTS OF THE ESTIMATED BIP VECTOR** $\hat{\bar{\pi }}$**, DAMPING** ${\hat{\bar{D}}}_{q}$**, AND STIFFNESS** ${\hat{\bar{K}}}_{q}$											
**Method**	${\hat{\bar{\pi }}}_{1}[\%]$	${\hat{\bar{\pi }}}_{2}[\%]$	${\hat{\bar{\pi }}}_{3}[\%]$	${\hat{\bar{D}}}_{q,11}[\%]$	${\hat{\bar{D}}}_{q,12}[\%]$	${\hat{\bar{D}}}_{q,21}[\%]$	${\hat{\bar{D}}}_{q,22}[\%]$	${\hat{\bar{K}}}_{q,11}[\%]$	${\hat{\bar{K}}}_{q,12}[\%]$	${\hat{\bar{K}}}_{q,21}[\%]$	${\hat{\bar{K}}}_{q,22}[\%]$
FJI	0.94	1.44	1.79	9.91 (1.48)	3.59 (1.29)	3.99 (1.03)	4.64 (0.91)	10.21 (6.30)	6.23 (4.17)	6.23 (4.17)	6.21 (3.82)
GOM	0.95	1.43	1.76	9.27 (1.99)	3.56 (1.52)	3.53 (1.43)	3.45 (1.05)	20.22 (9.76)	12.53 (6.23)	11.97 (7.01)	11.94 (5.57)

**Table:** 

**(B) RSSS, AICS, AND BICS OF THE VARIATIONAL EXTERNAL TORQUES** $\Delta \tau_{\text{ext}}$			
**Method**	**RSS [Nm^2^]**	**AIC [−]**	**BIC [−]**
FJI	8.10 (5.19)	16.38 · 10^3^ (6.13 · 10^3^)	16.45 · 10^3^ (6.13 · 10^3^)
GOM	17.31 (10.00)	23.58 · 10^3^ (5.90 · 10^3^)	23.66 · 10^3^ (5.90 · 10^3^)

***(A)** Mean (SD) results of the NAEs of the elements of the estimated BIP vector $\hat{\bar{\pi }}$, damping ${\hat{\bar{D}}}_{q}$, and stiffness ${\hat{\bar{K}}}_{q}$. **(B)** Mean (SD) results of the RSSs, AICs, and BICs of the variational external torques Δ**τ**_ext_ obtained from a simulation with the estimated BIP vector $\hat{\bar{\pi }}$, damping ${\hat{\bar{D}}}_{q}$, and stiffness ${\hat{\bar{K}}}_{q}$ (within the estimation interval [0, T_est_])*.

#### 3.2.2. Comparison

The NAEs of the elements of the estimated BIP vector $\hat{\bar{\pi }}$ of GOM are almost identical to those of FJI. The mean NAEs of the elements of the estimated damping ${\hat{\bar{D}}}_{q}$ of GOM are marginally smaller than those of FJI and the opposite applies to the corresponding SDs which essentially makes these NAEs almost identical as well. In contrast, a considerable difference in estimation accuracy is found in the elements of the estimated stiffness ${\hat{\bar{K}}}_{q}$, for which both the mean NAEs and the SDs of GOM are larger than those of FJI, with the mean NAEs being approximately twice as large for GOM as they are for FJI. These results are plausible, as the difference in estimation accuracy of the variational dynamics $\left\{\Delta x,\Delta \dot{x},\Delta \ddot{x},\Delta u_{\text{ext}}\right\}$ is largest for the variational positions Δ***x***. According to the involuntary impedance model, the difference in estimation accuracy of the variational angles Δ***q*** directly influences the estimated stiffness ${\hat{\bar{K}}}_{q}$.

The mean (SD) results of the RSSs, AICs, and BICs of the variational external torques Δ***τ***_ext_ are presented in Table 2B. FJI outperforms GOM in all three performance criteria. The difference in RSS is especially relevant, as it demonstrates, that the differences in AIC and BIC do not arise solely due to differences in the number of parameters *p*. Similar to the mean NAEs of the elements of the estimated stiffness ${\hat{\bar{K}}}_{q}$ in Table 2A, the mean RSSs are approximately twice as large for GOM as they are for FJI. The differences in RSS, AIC, and BIC demonstrate that (1) the differences in the estimated BIP vector $\hat{\bar{\pi }}$, damping ${\hat{\bar{D}}}_{q}$, and stiffness ${\hat{\bar{K}}}_{q}$, represented by the respective NAEs in Table 2A, have a substantial effect on the replicability of the real simulation output and that (2) the involuntary impedance estimation results of FJI yield a more accurate replication of the real simulation output than those of GOM.

### 3.3. Evaluation

In this section, we evaluate the performance of our method for real data obtained in an experiment with 12 human participants. For simplicity, from this point on, we omit the term estimated when referring to the results of the involuntary impedance estimation, i.e., BIP vector $\hat{\bar{\pi }}$, damping ${\hat{\bar{D}}}_{q}$, and stiffness ${\hat{\bar{K}}}_{q}$. In the experiment, every trial is either an unperturbed or a perturbed trial. Consequently, we can not evaluate the estimation accuracy of the unperturbed states. Therefore, we only evaluate the calculation of the cut-off frequencies ***f***_c,HP_ of the high pass filter.

#### 3.3.1. Feedback Jerk Isolation

**Figure 10:**
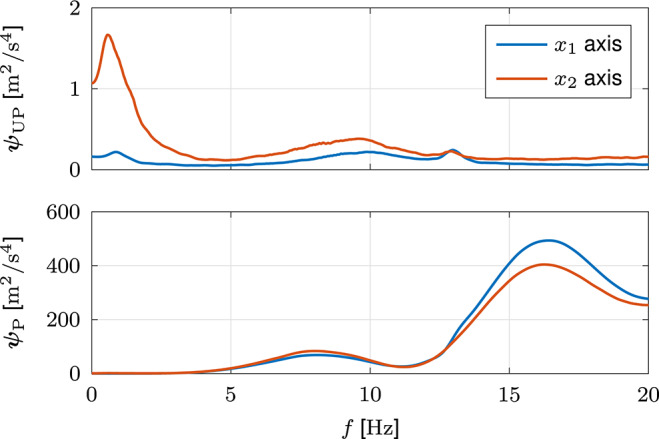
Filter configuration based on experimental data. Mean results of the ESDs ***ψ***_UP_ and ***ψ***_P_ of the jerks $\overset{⃛}{x}$ of the unperturbed and perturbed movements.

Figure 10 shows the mean results of the ESDs ***ψ***_UP_ and ***ψ***_P_, which look very similar to those of the simulation presented in Figure 7A. In the unperturbed movements of the experiment, the energy of the principal movement along the *x*_2_ axis is more widespread than in the simulation. However, the respective ESD ψ_UP,2_ nonetheless possesses a peak at a similar frequency. The ESD ψ_UP,1_ is significantly lower, due to the absence of movements along the *x*_1_ axis. Due to hardware noise, the energy of both axes of the unperturbed movements does not decrease to values as low as those of the simulation for high frequencies. However, the respective values are nonetheless significantly smaller than those of the perturbed movements, for which the energy increases to significantly higher values for high frequencies. The respective ESDs ***ψ***_P_ are much higher than those of the unperturbed movements and have multiple peaks in the high frequency range. While the overall energy of the unperturbed movements is slightly decreased, the overall energy of the perturbed movements is increased compared to the simulation. This difference is largely caused by high frequency oscillations in the jerks $\overset{⃛}{x}$ of the perturbed movements which result from oscillations due to the perturbations paired with sensor noise and high gain PD control. Nonetheless, the between-subject mean (SD) results of the cut-off frequencies ***f***_c,HP_ = [1.45 (0.30), 2.09 (0.24)] Hz are very similar to those of the simulation, with both values being slightly lower due to the slight decrease in overall energy of the unperturbed movements. Nonetheless, the cut-off frequency *f*_c,HP,2_ is still sufficiently high to lie above the energy of the principal movement.

#### 3.3.2. Involuntary Impedance Estimation

**Figure 11:**
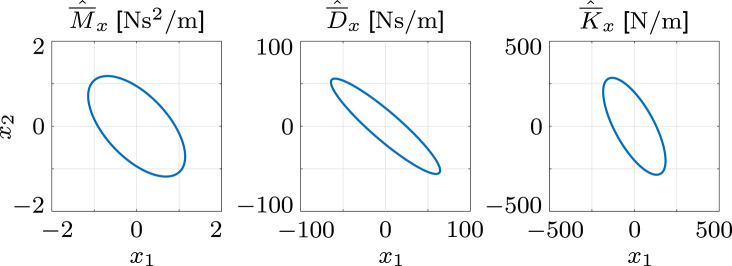
Evaluation of involuntary impedance estimation with experimental data. Cartesian endpoint ellipses of the mean inertia ${\hat{\bar{M}}}_{x}$, damping ${\hat{\bar{D}}}_{x}$, and stiffness ${\hat{\bar{K}}}_{x}$. The respective Cartesian space matrices are calculated by transformation of the BIP vector $\hat{\bar{\pi }}$, damping ${\hat{\bar{D}}}_{q}$, and stiffness ${\hat{\bar{K}}}_{q}$, respectively.

Table 3 lists the within- and cross-subject mean (SD) results of the involuntary impedance estimation. Analogous to the simulation, the elements of the BIP vector $\hat{\bar{\pi }}$ do not possess SDs, because they are estimated with the complete data set of the static task. The elements of the mean BIP vector $\hat{\bar{\pi }}$ are similar to the simulated values in the simulation ***π***_sim_ = [0.1945, 0.0737, 0.0838]. The first element ${\hat{\bar{\pi }}}_{1}$ of the BIP vector is decreased compared to the respective value in the simulation. As this element largely depends on the inertial characteristics of the upper arm, the difference could be caused by the comparatively little movement of the shoulder joint which results in less involvement of the upper arm. As the inertial parameters in Gomi and Kawato (1997) are defined by the linearized rigid body dynamics, the respective estimation results can not be used for comparison. Thus, we use the elements of the mean BIP vector $\hat{\bar{\pi }}$ to calculate the mean inertia ${\hat{\bar{M}}}_{q}$, which we transform to Cartesian space to obtain the mean Cartesian endpoint inertia ${\hat{\bar{M}}}_{x}$. The elements of this mean Cartesian endpoint inertia ${\hat{\bar{M}}}_{x}$ are similar to those reported in existing studies (Tsuji et al., 1995; Chang et al., 2013; Dyck and Tavakoli, 2013). Consequently, the same applies to the Cartesian endpoint ellipse, which is illustrated in Figure 11.

**Table 3:** Evaluation of involuntary impedance estimation with experimental data.

**#**	**$\underset{\left[{\text{kgm}}^{\text{2}}\right]}{{\hat{\bar{\pi }}}_{1}}$**	**$\underset{\left[{\text{kgm}}^{\text{2}}\right]}{{\hat{\bar{\pi }}}_{2}}$**	**$\underset{\left[{\text{kgm}}^{\text{2}}\right]}{{\hat{\bar{\pi }}}_{3}}$**	**$\underset{\left[\text{Nms/rad}\right]}{{\hat{\bar{D}}}_{q,11}}$**	**$\underset{\left[\text{Nms/rad}\right]}{{\hat{\bar{D}}}_{q,12}}$**	**$\underset{\left[\text{Nms/rad}\right]}{{\hat{\bar{D}}}_{q,21}}$**	**$\underset{\left[\text{Nms/rad}\right]}{{\hat{\bar{D}}}_{q,22}}$**	**$\underset{\left[\text{Nm/rad}\right]}{{\hat{\bar{K}}}_{q,11}}$**	**$\underset{\left[\text{Nm/rad}\right]}{{\hat{\bar{K}}}_{q,12/21}}$**	**$\underset{\left[\text{Nm/rad}\right]}{{\hat{\bar{K}}}_{q,22}}$**
01	0.0817	0.0582	0.0581	9.00 (0.19)	1.97 (0.08)	2.09 (0.13)	1.18 (0.16)	2.77 (1.92)	4.54 (2.61)	7.75 (4.03)
02	0.0890	0.0787	0.0780	8.68 (0.25)	2.19 (0.11)	2.47 (0.05)	1.92 (0.11)	33.66 (6.23)	1.24 (2.19)	16.34 (3.90)
03	0.1648	0.1021	0.0938	12.64 (0.23)	2.43 (0.12)	2.87 (0.06)	2.49 (0.04)	38.30 (11.12)	6.82 (2.99)	13.88 (4.17)
04	0.1074	0.0751	0.0714	9.08 (0.27)	2.01 (0.09)	2.10 (0.04)	1.56 (0.08)	12.46 (2.96)	13.43 (1.67)	15.40 (2.10)
05	0.0759	0.0877	0.0734	10.44 (1.01)	3.11 (0.36)	3.30 (0.20)	2.30 (0.14)	36.84 (23.14)	14.87 (4.28)	20.00 (3.95)
06	0.2099	0.0923	0.1076	12.70 (0.24)	2.14 (0.21)	2.86 (0.10)	1.98 (0.08)	67.15 (15.12)	18.19 (4.11)	17.62 (1.95)
07	0.1207	0.0677	0.0613	9.24 (0.21)	2.44 (0.26)	2.39 (0.10)	1.58 (0.07)	19.02 (10.65)	11.78 (8.73)	19.06 (5.76)
08	0.0887	0.0671	0.0692	8.07 (0.21)	2.08 (0.08)	2.13 (0.05)	1.72 (0.08)	30.30 (8.02)	1.04 (1.40)	7.60 (2.25)
09	0.2229	0.0838	0.1073	9.27 (0.55)	1.29 (0.19)	1.84 (0.12)	1.86 (0.05)	47.23 (34.71)	0.00 (0.00)	6.63 (1.04)
10	0.0777	0.0501	0.0509	6.61 (0.11)	1.28 (0.06)	1.28 (0.07)	1.03 (0.03)	2.50 (1.24)	4.68 (1.81)	8.96 (2.57)
11	0.1164	0.0918	0.0894	10.15 (0.35)	2.15 (0.04)	2.73 (0.02)	2.35 (0.05)	34.84 (7.62)	1.27 (1.37)	17.24 (1.35)
12	0.1558	0.0646	0.0702	8.15 (0.11)	1.32 (0.07)	1.74 (0.06)	1.34 (0.05)	14.58 (4.91)	7.31 (2.31)	10.55 (2.19)
Mean	0.1259	0.0766	0.0776	9.50 (0.31)	2.03 (0.14)	2.32 (0.08)	1.78 (0.08)	28.30 (10.64)	7.10 (2.79)	13.42 (2.94)

*Within- and cross-subject mean (SD) results of the elements of the estimated BIP vector $\hat{\bar{\pi }}$, damping ${\hat{\bar{D}}}_{q}$, and stiffness ${\hat{\bar{K}}}_{q}$*.

The elements of the mean damping ${\hat{\bar{D}}}_{q}$ are similar to the averaged simulated values *D*_*q*,sim_ = [2.42, 1.20, 1.20, 1.42], with the exception of the element ${\hat{\bar{D}}}_{q,11}$, which is comparatively large. As the element ${\hat{\bar{D}}}_{q,11}$ represents the contributions of the single-joint shoulder muscles, which are less involved due to less movement of the shoulder joint, it is more difficult to estimate. This correlation is also visible in the increased estimation error in the validation with simulated data (see Table 2A). As the remaining elements are decreased compared to the respective values in the simulation, it is possible that some of the contributions of these elements are allocated to the element ${\hat{\bar{D}}}_{q,11}$. As the damping results of Gomi and Kawato (1997) are not reported, we can not use them for comparison. However, the elements of the mean damping ${\hat{\bar{D}}}_{q}$ are of similar order of magnitude as those of static tasks (Tsuji et al., 1995; Lakatos et al., 2011). The overall increase in magnitude compared to the results of these static tasks is to be expected, as similar correlations are observed for estimates of stiffness (Gomi and Kawato, 1997).

The elements of the mean stiffness ${\hat{\bar{K}}}_{q}$ are similar to the averaged simulated values *K*_*q*,sim_ = [29.05, 14.37, 14.37, 17.08], with the elements ${\hat{\bar{K}}}_{q,12/21}$ being slightly decreased in comparison. Consequently, the elements of the mean stiffness ${\hat{\bar{K}}}_{q}$ are also similar to those reported for comparable dynamic tasks (Burdet et al., 2000; Darainy et al., 2007; Wong et al., 2009b), including those reported for the dynamic task in Gomi and Kawato (1997). The difference in size of the SDs of the stiffness ${\hat{\bar{K}}}_{q}$ and the damping ${\hat{\bar{D}}}_{q}$ fits to the difference in estimation errors in the validation with simulated data (see Table 2A). It could however also indicate that variations in damping during the course of the experiment are generally lower than those in stiffness or that some of the variations in damping are incorrectly interpreted as variations in stiffness by the non-linear least squares estimation.

**Figure 12:**
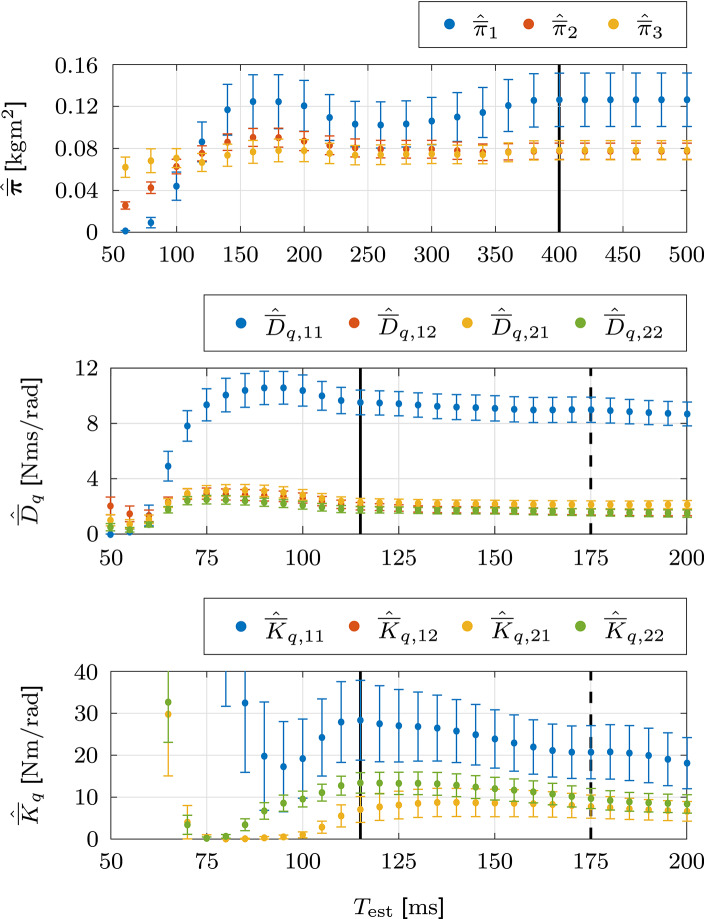
Evaluation of involuntary impedance estimation with experimental data. Between-subject mean (SD) results of the BIP vector $\hat{\bar{\pi }}$, damping ${\hat{\bar{D}}}_{q}$ and stiffness ${\hat{\bar{K}}}_{q}$ for different durations of the estimation interval *T*_est_. In order to avoid overlap, the error bars represent ±0.5between-subject SD. The solid vertical lines indicate the durations of the estimation intervals of the static task (*T*_est_ = 400ms) and the dynamic task (*T*_est_ = 115ms). The estimation intervals begin at the onset of the perturbations. The dashed vertical lines indicate the offset of the perturbations. For the static task, the duration of the estimation interval *T*_est_ and the offset of the perturbation, i.e., the solid and the dashed lines, coincide.

Figure 11 shows the Cartesian endpoint ellipses of the mean inertia ${\hat{\bar{M}}}_{x}$, damping ${\hat{\bar{D}}}_{x}$, and stiffness ${\hat{\bar{K}}}_{x}$. The shapes and orientations of the ellipses are similar to those in existing studies (Tsuji et al., 1995; Gomi and Osu, 1998; Darainy et al., 2007). Similar to the results for the movements along the sagittal axis in Gomi and Kawato (1997), the major axis of the Cartesian endpoint ellipse of the mean stiffness ${\hat{\bar{K}}}_{x}$ is oriented slightly more parallel to the *x*_2_ axis, i.e., the axis of the principal movement. Figure 12 shows the between-subject mean (SD) results of the BIP vector $\hat{\bar{\pi }}$, damping ${\hat{\bar{D}}}_{q}$, and stiffness ${\hat{\bar{K}}}_{q}$ for different durations of the estimation interval *T*_est_. In the static task, the elements of the BIP vector $\hat{\bar{\pi }}$ converge to constant values and do not change for durations *T*_est_ > 400 ms. Similar behavior is observable in the dynamic task for the elements of the damping ${\hat{\bar{D}}}_{q}$, with a slight decrease for durations *T*_est_ > 115 ms. In comparison, the elements of the stiffness ${\hat{\bar{K}}}_{q}$ converge at a slower rate and require a longer estimation interval to reach plausible values. Furthermore, the decrease for durations *T*_est_ > 115 ms is larger than that of the elements of the damping ${\hat{\bar{D}}}_{q}$. This decrease could be caused by the return to the unperturbed states. The longer the estimation interval, the larger the percentage of the variational data with small deviations from the unperturbed states. The larger this percentage becomes, the more influence the respective values of the elements of the stiffness ${\hat{\bar{K}}}_{q}$ have on the solution of the non-linear least squares estimation. The decrease could however also be caused by the activation of voluntary feedback contributions.

## 4. Discussion

The mean results of the involuntary impedance estimation for different durations *T*_est_ in Figure 12 show that the elements of the damping ${\hat{\bar{D}}}_{q}$ and the stiffness ${\hat{\bar{K}}}_{q}$ reach plausible values for the estimation interval with duration *T*_est_ = δ_v_ = 115 ms. While the elements of the damping ${\hat{\bar{D}}}_{q}$ already reach plausible values at *T*_est_ ≈ 75 ms, the amount of information necessary for estimation of plausible values of the elements of the stiffness ${\hat{\bar{K}}}_{q}$ is not reached until *T*_est_≈110 ms. The mean results in Figure 12 show that the elements of the BIP vector $\hat{\bar{\pi }}$ converge to plausible values for the estimation interval with duration *T*_est_ = 400 ms. These results, in combination with the mean (SD) results in Table 3, which are similar to those reported for comparable dynamic tasks, successfully demonstrate the applicability of our method to real data.

The mean (SD) results of the NRMSEs in Table 1A show that our feedback jerk isolation achieves higher estimation accuracy for the variational dynamics $\left\{\Delta \hat{x},\Delta \dot{\hat{x}},\Delta \ddot{\hat{x}},\Delta {\hat{u}}_{\text{ext}}\right\}$ than the methods reported in Gomi and Kawato (1997) and Erden and Billard (2015). The difference in estimation accuracy is especially large for the variational positions $\Delta \hat{x}$. As a consequence, the estimation performance of the involuntary impedance estimation is increased for the elements of the stiffness ${\hat{\bar{K}}}_{q}$, which is shown by the mean (SD) results of the NAEs in Table 2A. The mean (SD) results of the NRMSEs for different simulation configurations in Tables 1B–D show that the estimation accuracy of the feedback jerk isolation is decreased for a higher movement velocity. This is plausible, as a higher movement velocity results in an increased cut-off frequency of the high pass filter which causes an increased information loss in the isolation of the feedback jerk. Due to similar reasons, a higher cut-off frequency of the neural noise also leads to a decrease of the estimation accuracy. For all of the remaining simulation configurations, changes in the estimation accuracy are marginal. While the estimation accuracy of the method in Gomi and Kawato (1997) is similarly decreased for a higher cut-off frequency of the neural noise, it is contrastly increased for a higher movement velocity. As this method depends on the similarity of the movement to the averaged unbiased dynamics, an increase of the amplitude of the neural noise leads to a significant decrease of estimation accuracy, especially in the variational positions $\Delta \hat{x}$. In summary, while the feedback jerk isolation is outperformed by the method in Gomi and Kawato (1997) for movements with high velocity and low movement variance due to neural noise, it provides superior estimation performance for movements with moderate to low velocity and moderate to high movement variability due to neural noise, as it is much less affected by a decrease in the similarity of the movements.

According to the main ISO safety standard for robots (DIN EN ISO 10218-1:2011), the maximum robot end-effector velocity during collaboration with a human must not exceed 250 mm/s (Colgate et al., 2008). Some studies on safe physical human-robot collaboration use more conservative values for the maximum robot end-effector velocity, e.g., 150 mm/s in Neranon (2020) and 100 mm/s in Weitschat et al. (2018). In the simulated data used to obtain the NRMSEs in Table 1B, the mean (SD) peak velocities *ẋ*_peak_ that correspond to the different durations of the movements *T*_mov_ are *ẋ*_peak_(*T*_mov_ = 1 s) = 747.8 (6.3) mm/s, *ẋ*_peak_(*T*_mov_ = 2 s) = 377.6 (8.9) mm/s, and *ẋ*_peak_(*T*_mov_ = 3 s) = 254.0 (9.8) mm/s. Thus, the peak velocities *ẋ*_peak_ of those movements, for which the method in Gomi and Kawato (1997) outperforms the feedback jerk isolation, are far above the current constraints for safe pHRI. For those movements with peak velocities *ẋ*_peak_ that are similar to or moderately increased compared to the current constraints, the feedback jerk isolation provides superior estimation performance. As the neural noise parameters in the simulation are calculated to provide movement variability similar to that observed in repetitive, straight reaching movements (Burdet et al., 2001) and both neural and kinematic variability are shown to be interrelated individual characteristics (Haar et al., 2017), it is unlikely that realistic pHRI scenarios possess a lower amount of movement variability. Thus, based on these circumstances, we conclude that the feedback jerk isolation is well-suited for our envisaged application in realistic pHRI scenarios.

We approximate the feedback behavior by a linearized model. This approximation is analogously applied in comparable studies (Dolan et al., 1993; Burdet et al., 2000; Darainy et al., 2007) that include deviations of similar size or larger than the ones in our work. In our static task, the position perturbations with amplitudes of 8 mm result in mean (SD) maximum external force deviations of 4.75 (1.80) N. In our dynamic task, the force perturbations result in mean (SD) maximum position deviations of 3.53 (0.34) mm and external force deviations of 9.48 (3.05) N. The mean (SD) results of the NAEs in Table 2A shows that the estimation errors of the elements of the BIP vector $\hat{\bar{\pi }}$ are almost identical for the dynamic regressor representation and the linearized rigid body dynamics of Gomi and Kawato (1997). This indicates that the variational dynamics $\left\{\Delta q,\Delta \dot{q},\Delta \ddot{q},\Delta \tau_{\text{ext}}\right\}$ of the static task are sufficiently small to allow for linearization of the rigid body dynamics without loss of estimation accuracy and supports the assumption that the feedback behavior evoked by the force perturbations can be approximated by a linearized model.

Another advantage of perturbations with comparatively small amplitudes is that they are less likely to lead to instability of the movement than those with larger amplitudes. The region of stability of the movement is influenced by a number of factors, including feedforward and voluntary feedback behavior, which are both highly task-specific. As stiffness is positively correlated with internal torques, movements that require larger feedforward torques, e.g., due to increased movement velocity or interaction forces, coincide with larger stiffness (Tee et al., 2004). Thus, for these movements, perturbations with a certain amplitude result in smaller deviations. Assuming that the voluntary feedback behavior can be modeled by stochastic optimal control, the effects of the perturbations after the delay of voluntary feedback depend on the incorporated cost functions, e.g., defined by the tracking error or the smoothness of the movement (Todorov and Jordan, 2002). Furthermore, the region of stability is also heavily influenced by the level of muscle cocontraction, which is known to increase in response to a number of factors, e.g., incomplete or incorrect internal models (Tomi et al., 2008) and increased accuracy requirements (Lametti et al., 2007). In Burdet et al. (2001), participants increase their level of muscle cocontraction in order to successfully compensate effects of unpredictable environmental instabilities. Due to all of these factors, assessment of the region of stability must take into consideration task-specific influences and constraints. In future work, we aim to apply our method to more complex movements and complement corresponding assessments of the region of stability, e.g., in the form of numerical assessments based on Monte Carlo methods.

Multiple studies present impedance estimation methods based on Kalman filters (Deng et al., 2006; Asao et al., 2013) or extended Kalman filters (Roveda et al., 2013). The advantage of these filters is that, given continuous observations of the relevant variables, they allow for estimation of time-varying values of damping and stiffness. However, their application requires a model of the complete system dynamics, including feedforward and voluntary feedback behavior (if the respective method is used for a duration that is longer than the delay of voluntary feedback). Some studies avoid these limitations by assuming that the combination of feedforward and feedback behavior can be modeled by the sum of damping and stiffness (Asao et al., 2013) or just stiffness (Roveda et al., 2013). In Deng et al. (2006), the authors assume that effects of feedforward and voluntary feedback behavior are neglectable due to application of pseudo-random perturbations in combination with a band pass filter. However, the plausibility of these assumptions is not validated, as the method is only applied to a simulation that models the combination of feedforward and feedback behavior by the sum of damping and stiffness. Thus, existing impedance estimation methods based on Kalman filters are extremely limited in possible application scenarios and the application to realistic pHRI would require significantly more complex models. While our method is not able to provide time-varying values of damping and stiffness, it is able to provide accurate estimates within a limited interval without the need for modeling feedforward and voluntary feedback behavior.

## 5. Conclusion

In this work, we present a method for the estimation of the involuntary impedance components of the human arm during multi-joint movements by application of force perturbations. These perturbations are designed such that the evoked feedback jerk frequency content can be isolated with a high pass filter. We limit the duration of the estimation interval to 115 ms to guarantee exclusion of voluntary feedback. We estimate the inertial parameters in a static postural task and subsequently insert them to estimate the damping and stiffness in a dynamic movement task.The evaluation of the experimental data shows that our method is able to provide plausible involuntary impedance estimates within the limited estimation interval that guarantees exclusion of voluntary feedback. Furthermore, the validation with simulated data shows that it provides superior estimation performance compared to the results obtained by application of existing methodologies within identical conditions. As the difference in estimation accuracy is especially large for the variational positions, the estimation performance is increased for the elements of the stiffness. The analysis of different movement velocities and variations of neural noise shows that the feedback jerk isolation is able to provide superior estimation performance for movements with moderate to low velocity and is much less affected by an increase in movement variability. We conclude that our method allows for involuntary impedance estimation in experiments that emulate realistic pHRI, without the need to include the do-not-intervene-voluntarily paradigm or comparable constraints within the respective dynamic movement tasks. This enables the acquisition of valuable information regarding involuntary impedance modulation strategies, which could be used for assessment of stability and adaptation of robot control behavior during pHRI. In future work, we aim to apply the method to more complex movements, inspired by realistic pHRI scenarios. Furthermore, we intend to complement our current apparatus by EMG sensing modalities, in order to obtain additional insights into the involved muscle activations.

## Data Availability Statement

The raw data supporting the conclusions of this article will be made available by the authors, without undue reservation, to any qualified researcher.

## Ethics Statement

The studies involving human participants were reviewed and approved by Ethics committee at the Technical University of Munich. The patients/participants provided their written informed consent to participate in this study. Written informed consent was obtained from the individual(s) for the publication of any potentially identifiable images or data included in this article.

## Author Contributions

HB and SE adapted the simulation and validated the method with simulated data. HB implemented and conducted the experiment, verified the method with experimental data, and wrote the first draft of the manuscript. SE and SH provided the critical revisions. All authors contributed to the conceptualization and methodology, read and approved the manuscript.

## Conflict of Interest

The authors declare that the research was conducted in the absence of any commercial or financial relationships that could be construed as a potential conflict of interest.

## Footnotes

^1^Due to the inclusion of reflexive feedback, according to the terminology established in Latash and Zatsiorsky (1993), the matrices *D*_*q*_ and *K*_*q*_ represent apparent impedance components. In this work, in order to highlight the exclusion of voluntary feedback, we refer to them as involuntary impedance components. For simplicity, we refer to the individual matrices *D*_*q*_ and *K*_*q*_ as damping and stiffness instead of involuntary joint damping and stiffness.
